# Machine Learning–Derived Cardiovascular Aging Phenotypes From Cardiac Function and Stroke Risk in the UK Biobank: Cohort Study

**DOI:** 10.2196/77017

**Published:** 2026-04-27

**Authors:** Kang Yuan, Deyan Kong, Jinghui Zhong, Mengdi Xie, Rui Liu, Wen Sun, Xinfeng Liu

**Affiliations:** 1 Department of Neurology, Affiliated Jinling Hospital, Medical School of Nanjing University, 305 Zhongshan East Road, Xuanwu District, Nanjing, Jiangsu Province, 210002, China, 86 2584801861, 86 2584805169; 2 Department of Neurology, The Second Affiliated Hospital of Guangxi Medical University, Nanning, Guangxi, China; 3 Department of Neurology, Centre for Leading Medicine and Advanced Technologies of IHM, The First Affiliated Hospital of USTC, Division of Life Sciences and Medicine, University of Science and Technology of China, Hefei, Anhui, China

**Keywords:** stroke, generative topographic mapping, UK Biobank, machine learning, cardiac function, aging, phenotypes

## Abstract

**Background:**

Cardiovascular magnetic resonance (CMR) is widely used across various cardiac conditions and systematically assesses cardiac anatomical structures and functional dynamics. Machine learning (ML) can accurately predict outcomes and understand the inherent features of clinical data.

**Objective:**

This study aimed to derive CMR phenotypes related to cardiovascular aging, investigate the relationship between these phenotypes and stroke risk, and relearn these phenotypes using supervised ML.

**Methods:**

We enrolled 36,467 participants without stroke and extracted CMR parameters from the UK Biobank, with follow-up data extending until September 30, 2023. Using the generative topographic mapping technique, we identified latent grid nodes among participants and then derived phenotypes through agglomerative hierarchical clustering. We used supervised ML models to predict cardiac function phenotypes and used Cox proportional hazards models to assess the association between these phenotypes and long-term stroke risk.

**Results:**

We enrolled 36,467 participants in the study. The mean age was 54.9 (SD 7.5) years, with 17,442 (47.8%) male participants. During a mean follow-up time of 14.7 (SD 1.1) years, 500 (1.4%) participants developed stroke and 664 (1.8%) participants died, respectively. After generative topographic mapping modeling, we identified 2 distinct phenotypes: phenotype 1, characterized by adverse cardiac function and an accumulation of cardiovascular risk factors, reflecting cardiovascular aging; and phenotype 2, associated with a lower risk of stroke (hazard ratio 0.695, 95% CI 0.559-0.864; *P*=.001), which remained significant after accounting for competing mortality (hazard ratio 0.578, 95% CI 0.484-0.691; *P*<.001). We selected the random forest model as the optimal model for the phenotypes, demonstrating high accuracy (area under the curve 0.914, 95% CI 0.911-0.918 for training and 0.867, 95% CI 0.858-0.876 for validation) and calibration ability (Brier score 0.111, 95% CI 0.109-0.113 for training and 0.132, 95% CI 0.127-0.137 for validation).

**Conclusions:**

By integrating unsupervised and supervised ML methods, we identified cardiovascular aging–related phenotypes that demonstrate robust predictive ability for incident stroke, which may have the potential to improve preventive and therapeutic strategies for high-risk populations.

## Introduction

Stroke is a major cause of mortality and morbidity worldwide [1]. Aging is associated with progressive structural and functional deterioration of the cardiovascular system [2]. Several conditions associated with cardiovascular aging [3,4], including atrial fibrillation, heart failure, and myocardial infarction, are recognized as important risk factors for stroke [5-8]. Cardiovascular magnetic resonance (CMR) enables systematic assessment of cardiac structural and functional remodeling and has been reported to be associated with the aging process [9]. Previous studies have shown that atrial mechanical dysfunction and left ventricular (LV) structural abnormalities assessed by CMR are associated with stroke events [10,11]. However, CMR metrics are relatively complex, and extracting phenotypes from these metrics may provide insight into their relationship with cardiovascular aging and help clarify their association with stroke risk.

The application of artificial intelligence in clinical practice for disease prediction and detection has grown tremendously in recent years [12,13]. Supervised machine learning (ML) can accurately predict clinical outcomes, while unsupervised ML can identify the inherent features of data without requiring labeled outcomes [14]. Previous studies have used unsupervised ML models to delineate prognostically distinct clusters using variables related to LV diastolic function derived from transthoracic echocardiography [15]. Additionally, the hierarchical k-means clustering algorithm has also been effective in distinguishing potential embolic source groups in cryptogenic stroke [16]. Generative topographic mapping (GTM) is a probabilistic model used for dimensionality reduction and data visualization, exhibiting superiority over the self-organizing map algorithm by explicitly modeling probability distributions [17,18]. GTM can reduce data dimensionality to a latent space and reveal the underlying structure with robustness across diverse sources [19]. However, previous studies applying GTM to derive phenotypes have been limited, with applications reported in general and critical care populations [20].

Therefore, the aim of this study was to use the GTM model to identify distinct cardiac function phenotypes related to cardiovascular aging, examine their association with long-term stroke risk, and develop supervised ML models to predict these phenotypes using data from a prospective longitudinal cohort.

## Methods

### Study Population

The UK Biobank is a large, population-based prospective cohort study comprising more than 500,000 participants aged 49 to 69 years, enrolled from 22 assessment centers in the United Kingdom between 2006 and 2010. Details of the UK Biobank protocols are accessible online [21]. Participants diagnosed with stroke at baseline (n=7780, 1.5%), lacking follow-up information (n=20,251, 4%), or without CMR data (n=437,713, 87.2%) were excluded, resulting in a final cohort of 36,467 (7.3%) participants.

**Figure 1.**
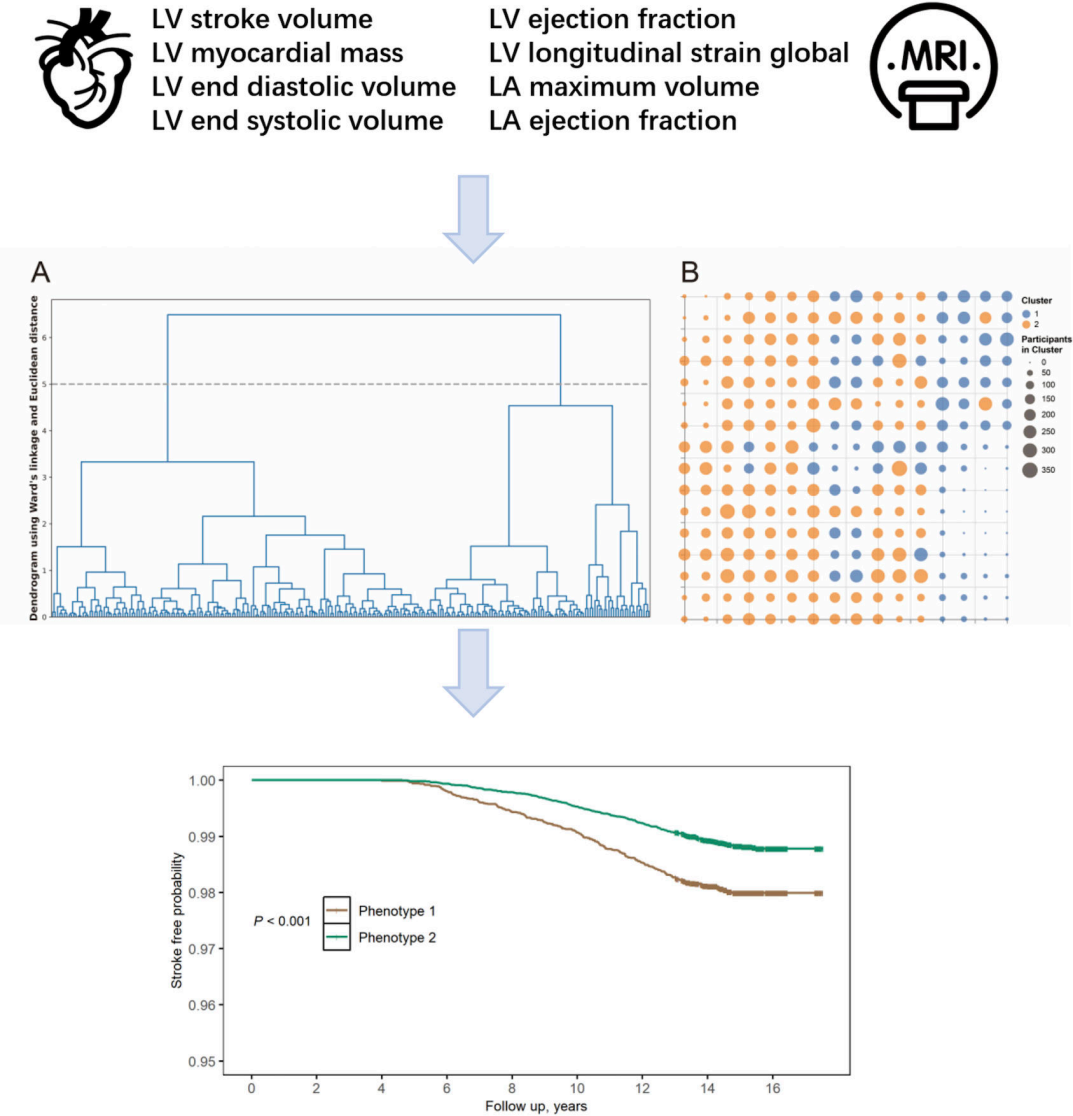
Cardiovascular aging phenotypes and stroke. LA: left atrial; LV: left ventricular.

We included the following CMR measures in the study: LV stroke volume (LVSV), LV myocardial mass (LVMM), LV end-diastolic volume (LVEDV), LV end-systolic volume (LVESV), LV ejection fraction (LVEF), LV global longitudinal strain (LVGLS), left atrial (LA) maximum volume (LAMV), and LA ejection fraction (LAEF) (Figure 1).

Stroke diagnosis was ascertained through linkage to self-reported medical conditions, hospital inpatient data, and death register records based on *International Classification of Diseases* codes, with the earliest recorded date of outcome provided in Table S1 in Multimedia Appendix 1. The follow-up time was defined as the interval from the date of recruitment to the occurrence of stroke, all-cause mortality, loss to follow-up, or September 30, 2023, whichever came first. Details of covariates were shown in Table S2 in Multimedia Appendix 1. This study was reported according to the transparent reporting of a multivariable prediction model for individual prognosis or diagnosis (TRIPOD) guidelines (Checklist 1).

### GTM Modeling and Phenotypes

Inspired by the algorithm introduced by Bellfield et al [20], we used a Gaussian mixture model to train the GTM model and visualize the distribution of data in the latent space. GTM uses a soft assignment method to estimate the probability of each participant being assigned to a cluster. The high-dimensional data were then visualized in a 2D latent space. After preprocessing the dataset, we used the *ugtm* Python (Python Software Foundation) package for GTM modeling and applied 10-fold cross-validation with the expectation-maximization algorithm to optimize the parameters. After GTM modeling, the generated latent space illustrated the distribution of participants in each cluster, with darker colors indicating higher densities. The modeling parameters (cardiac function variables) demonstrated the impact of cardiac function on each latent grid node in the GTM model. We aggregated the latent grid nodes by calculating the Euclidean distances between their modeling parameters and applied agglomerative hierarchical clustering using the Ward minimum variance method to derive phenotypes [22]. The characteristics of the phenotypes were compared to evaluate the distribution of risk factors for cardiovascular aging.

### Phenotypes and Stroke Risk

We used Cox proportional hazards models to evaluate the association between cardiac function phenotypes and long-term stroke risk, reporting results as hazard ratios (HRs) with 95% CIs. In multivariable analyses, model 1 was an unadjusted model; model 2 was adjusted for age at recruitment, sex, Townsend deprivation index at recruitment, smoking status, alcohol intake frequency, systolic blood pressure, diastolic blood pressure, and BMI; and model 3 was additionally adjusted for atrial fibrillation, type 2 diabetes, coronary heart disease, heart failure, antihypertensive drugs, and statins. In sensitivity analyses, we explored the association of cardiac function phenotypes with the risk of different stroke types. Additionally, we performed a competing risk analysis for stroke risk using the Fine and Gray method, accounting for mortality as the competing event.

### Supervised ML Frameworks

Following the categorization of participants into different phenotypes through the GTM approach, we deployed several supervised ML models to predict cardiac function phenotypes. Hyperparameters for each model are detailed in Table S3 in Multimedia Appendix 1. Hyperparameter optimization was conducted using grid search and Bayesian optimization. Discriminatory ability was evaluated using the receiver operating characteristic curve. Calibration performance was assessed using the calibration curve. We selected the best-performing method as the final algorithm for developing a phenotype prediction model. We constructed a website using the Flask framework and the DeepSeek-R1 API (application programming interface). The predicted probabilities and individualized metrics were sent to the DeepSeek-R1 model to generate a detailed analysis of health status and provide professional recommendations. Detailed descriptions of the methods are provided in Multimedia Appendix 1.

### Ethical Considerations

The ethical framework of the UK Biobank was approved by the North West Multi-centre Research Ethics Committee (11/NW/0382). All participants provided informed consent through electronic signature at enrollment.

### Statistical Analysis

Normally distributed continuous data are presented as mean (SD), and group comparisons were performed using 2-tailed *t* tests. Categorical data were presented as frequency (percentage), and group comparisons were conducted using chi-square tests. To account for multiple comparisons, *P* values were adjusted using the false discovery rate method. Statistical tests were conducted with R software (version 4.2.1; R Foundation for Statistical Computing), and a 2-sided *P* value <.05 was considered statistically significant.

## Results

### Study Population

**Figure 2.**
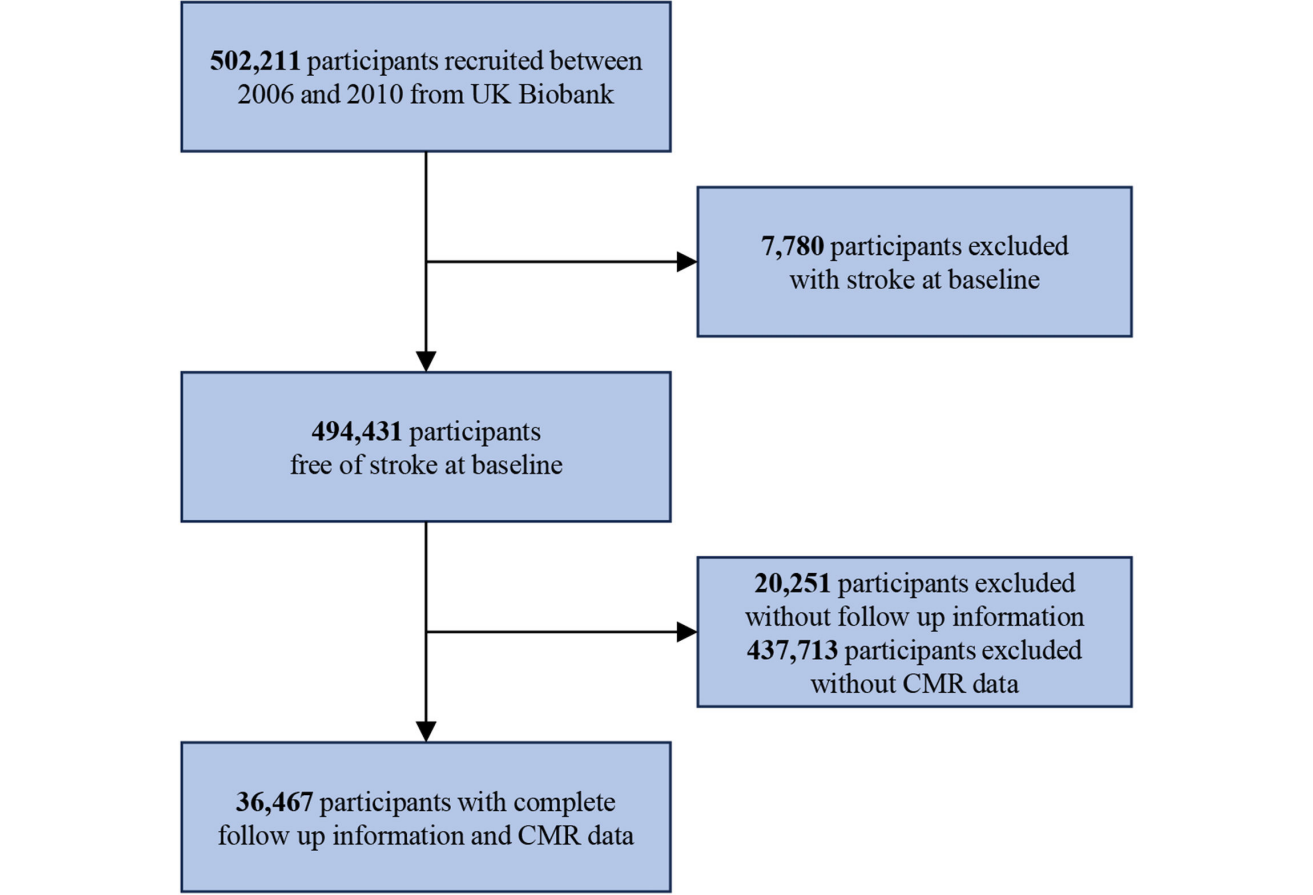
Flowchart illustrating the study design. CMR: cardiovascular magnetic resonance.

The study design is shown in Figure 2. Of the 36,467 participants included in the study (Figure 2), the mean age was 54.9 (SD 7.5) years, with 17,442 (47.8%) male participants. In total, 14,373 (39.5%) of participants were current or former smokers, 8180 (22.4%) had daily or almost daily alcohol intake, 1047 (2.9%) had type 2 diabetes, 345 (0.9%) had atrial fibrillation, and 48 (0.1%) had heart failure at baseline. During a mean follow-up time of 14.7 (SD 1.1) years, 500 (1.4%) and 664 (1.8%) participants developed stroke and died, respectively (Table 1).

**Table 1.** Characteristics of the participants included in the study.

Characteristics	Total (n=36,467)	Phenotype 1 (n=10,749)	Phenotype 2 (n=25,718)	Chi square (*df*) or t test (*df*)	*P* value	Adjusted *P* value^a^
Age (years), mean (SE)	54.9 (0.0)	54.4 (0.1)	55 (0.0)	−6.74 (19,563.5)^b^	<.001	<.001
Male, n (%)	17,442 (47.8)	9333 (86.8)	8109 (31.5)	9287 (1)^c^	<.001	<.001
Ethnicity, n (%)				78.9 (4)^c^	<.001	<.001
Asian	367 (1.0)	45 (0.4)	322 (1.3)			
Black	219 (0.6)	76 (0.7)	143 (0.6)			
Chinese	102 (0.3)	9 (0.1)	93 (0.4)			
Mixed	162 (0.4)	40 (0.4)	122 (0.5)			
White^d^	35,342 (97.7)	10,503 (98.4)	24,839 (97.3)			
Townsend deprivation index, mean (SE)	−1.9 (0.0)	−2 (0.0)	−1.9 (0.0)	−2.39 ( 20,325.8)^b^	.02	.02
Overall health rating, n (%)				35.6 (3)^c^	<.001	<.001
Poor	629 (1.7)	199 (1.9)	430 (1.7)			
Fair	5123 (14.1)	1683 (15.7)	3440 (13.4)			
Good	22,137 (60.8)	6359 (59.2)	15,778 (61.5)			
Excellent	8503 (23.4)	2492 (23.2)	6011 (23.4)			
Smoking status, n (%)				88.8 (2)^c^	<.001	<.001
Current	2297 (6.3)	771 (7.2)	1526 (5.9)			
Previous	12,076 (33.2)	3859 (36.0)	8217 (32.0)			
Never	22,007 (60.5)	6089 (56.8)	15,918 (62.0)			
Alcohol intake frequency, n (%)				415 (5)^c^	<.001	<.001
Daily or almost daily	8180 (22.4)	2800 (26.1)	5380 (20.9)			
3 or 4 times a week	10,190 (28.0)	3339 (31.1)	6851 (26.7)			
Once or twice a week	9389 (25.8)	2712 (25.2)	6677 (26.0)			
1-3 times a month	3963 (10.9)	946 (8.8)	3017 (11.7)			
Special occasions only	2997 (8.2)	554 (5.2)	2443 (9.5)			
Never	1728 (4.7)	393 (3.7)	1335 (5.2)			
Comorbidity, n (%)						
Dementia	8 (0.0)	2 (0.0)	6 (0.0)	0.001 (1)^c^	.99	.99
Major adverse cardiac events	857 (2.4)	432 (4.0)	425 (1.7)	184 (1)^c^	<.001	<.001
Type 2 diabetes	1047 (2.9)	368 (3.4)	679 (2.6)	16.4 (1)^c^	<.001	<.001
Liver disease	416 (1.1)	118 (1.1)	298 (1.2)	0.199 (1)^c^	.66	.73
Renal disease	446 (1.2)	132 (1.2)	314 (1.2)	0.001 (1)^c^	.99	.99
Atrial fibrillation	345 (0.9)	212 (2.0)	133 (0.5)	170 (1)^c^	<.001	<.001
Heart failure	48 (0.1)	35 (0.3)	13 (0.1)	41.6 (1)^c^	<.001	<.001
Coronary heart disease	1090 (3.0)	528 (4.9)	562 (2.2)	193 (1)^c^	<.001	<.001
Venous thrombosis	471 (1.3)	141 (1.3)	330 (1.3)	0.029 (1)^c^	.87	.91
Abdominal aortic aneurysm	5 (0.0)	3 (0.0)	2 (0.0)	1.01 (1)^c^	.31	.39
Peripheral arterial disease	255 (0.7)	68 (0.6)	187 (0.7)	0.844 (1)^c^	.36	.44
Cardiac function, mean (SE)^c^					<.001	<.001
Left ventricular stroke volume (mL)	87.5 (0.1)	104 (0.2)	80.6 (0.1)	113 (15,816.1)^b^		
Left ventricular myocardial mass (g)	86.1 (0.1)	109.2 (0.2)	76.4 (0.1)	161 (17,230.0)^b^		
Left ventricular end-diastolic volume (mL)	147.9 (0.2)	184.7 (0.3)	132.5 (0.1)	171 (16,189.1)^b^		
Left ventricular end-systolic volume (mL)	60.5 (0.1)	80.7 (0.2)	52 (0.1)	149 (14,438.1)^b^		
Left ventricular ejection fraction (%)	59.5 (0.0)	56.3 (0.1)	60.9 (0.0)	−64.2 (17,086.9)^b^		
Left ventricular longitudinal strain global (%)	−18.5 (0.0)	−17.3 (0.0)	−19 (0.0)	55 (19,909.5)^b^		
Left atrial maximum volume (mL)	72.8 (0.1)	92.6 (0.2)	64.4 (0.1)	109 (15,314.3)^b^		
Left atrial ejection fraction (%)	61.2 (0)	56.5 (0.1)	63.1 (0.1)	−59.8 (17,184.7)^b^		
Outcomes					<.001	
Follow-up time (y), mean (SE)	14.7 (0.0)	14.6 (0.0)	14.7 (0.0)	−3.05 (19,080.5)^b^		.003
Stroke, n (%)	500 (1.4)	209 (1.9)	291 (1.1)	36.4 (1)^c^		<.001
Mortality, n (%)	664 (1.8)	265 (2.5)	399 (1.6)	34.9 (1)^c^		<.001

a *P* values were adjusted by false discovery rate.

b *t* test.

c Chi-square test.

d Missing values for this row: n=275 missing for the Total column, n=76 missing for the Phenotype 1 column, and n=199 missing for the Phenotype 2 column.

### GTM Modeling and Cardiac Aging Phenotypes

**Figure 3.**
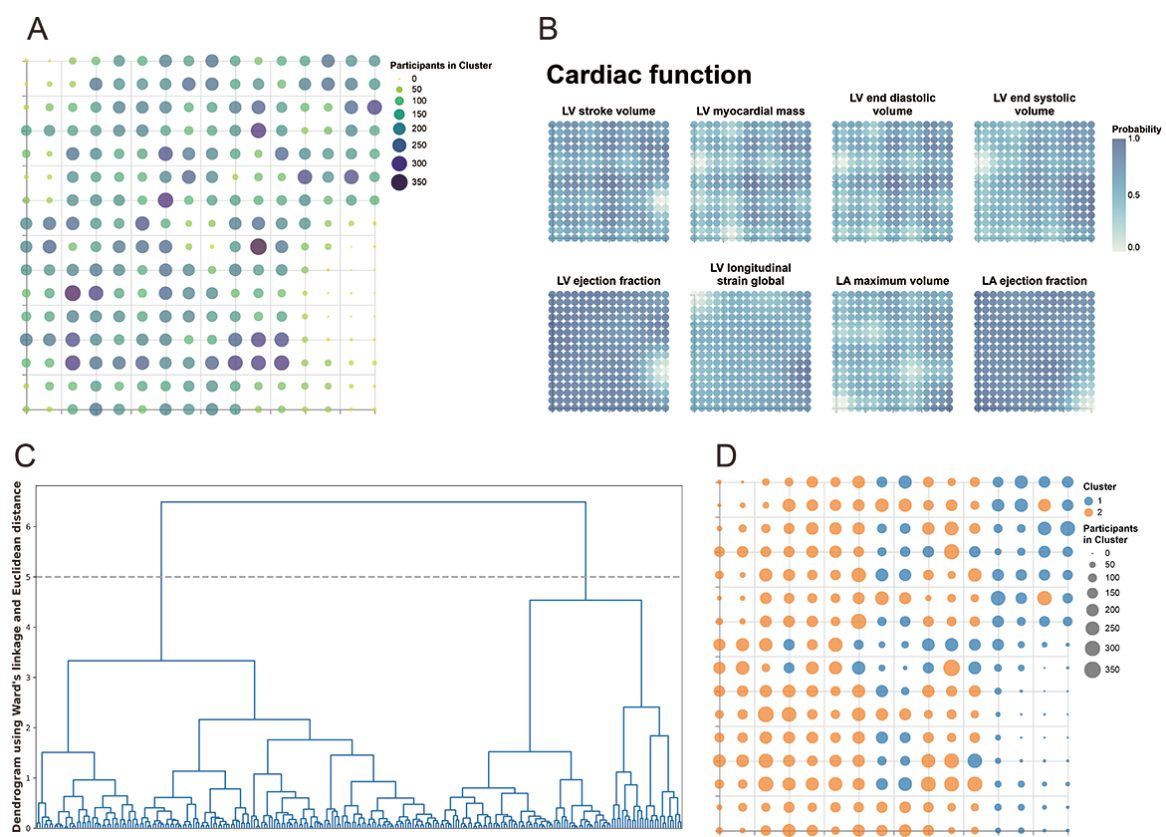
Derived phenotypes of cardiac function. (A) Latent space showing the distribution of participants; (B) latent space displaying the distribution of cardiac function markers derived from cardiovascular magnetic resonance; (C) dendrogram using Ward linkage and Euclidean distance to identify 2 cardiac function phenotypes; and (D) phenotypes and latent grid nodes of cardiac function. LA: left atrial; LV: left ventricular.

Figure 3 illustrates the latent space generated by the GTM model. The number of participants in clusters is represented using the “viridis” color scheme and visualized as circles, where a deeper blue hue and larger circle diameter indicate a higher number of participants. LVSV, LVMM, LVEDV, LVESV, LVEF, LVGLS, LAMV, and LAEF are aligned point-to-point with their latent space in a light teal blue scheme. In the dendrogram plot, 2 cardiac function phenotypes are identified, clearly separating the dataset into 2 major branches at a relatively large linkage distance. Figure S1 in Multimedia Appendix 1 illustrates that stroke diagnoses among participants were mainly distributed in the area of phenotype 1.

To further characterize the phenotype patterns, we compared the distributions of investigatory variables and visualized them on the latent space with the light orange color scheme (Figures S2-S7 in Multimedia Appendix 1). Significant differences were observed between the 2 cardiac function phenotypes across demographic characteristics, cardiovascular risk factors, and cardiac function parameters. Participants in phenotype 1 exhibited larger cardiac chamber volumes and greater myocardial mass, with higher LVEF and LAEF but lower LVSV, LVMM, LVEDV, LVESV, LVGLS, and LAMV compared with phenotype 2 (all *P*<.001). In addition, phenotype 1 demonstrated a higher burden of cardiovascular risk factors and comorbidities, including a greater prevalence of major adverse cardiac events, atrial fibrillation, heart failure, coronary heart disease, and type 2 diabetes (*P*<.001). Participants in phenotype 1 also had higher blood pressure, arterial stiffness index, and more adverse metabolic profiles. Taken together, these structural, functional, and clinical differences suggest that phenotype 1 represents a cardiovascular aging–related phenotype, characterized by cardiac remodeling, increased cardiovascular risk burden, and elevated incidence of stroke and mortality (Table 1 and Table S4 in Multimedia Appendix 1).

### Phenotypes and Stroke Risk

Multivariable modeling identified that cardiovascular aging phenotypes were associated with the long-term risk of stroke and mortality. Compared with phenotype 1, phenotype 2 was significantly associated with a decreased risk of stroke in the unadjusted model (HR 0.579, 95% CI 0.484-0.691; *P*<.001); model 2 (HR 0.671, 95% CI 0.541-0.833; *P*<.001); and model 3 (HR 0.695, 95% CI 0.559-0.864; *P*=.001; Table 2).

**Table 2.** Association between cardiovascular aging phenotypes and long-term risk of stroke and mortality.

Outcome (phenotype 2 vs phenotype 1)	Stroke		Mortality		
	HR^a^ (95% CI)	*P* value	HR (95% CI)	*P* value	
Model 1^b^	0.579 (0.484-0.691)	<.001	0.625 (0.535-0.730)	<.001	
Model 2^c^	0.671 (0.541-0.833)	<.001	0.760 (0.632-0.913)	.003	
Model 3^d^	0.695 (0.559-0.864)	.001	0.772 (0.641-0.929)	.006	

a HR: hazard ratio.

b Model 1 was unadjusted.

c Model 2 was adjusted for age at recruitment, sex, Townsend deprivation index at recruitment, smoking status, alcohol intake frequency, systolic blood pressure, diastolic blood pressure, and BMI.

d Model 3 was additionally adjusted for atrial fibrillation, type 2 diabetes, coronary heart disease, heart failure, antihypertensive drugs, and statins.

**Figure 4.**
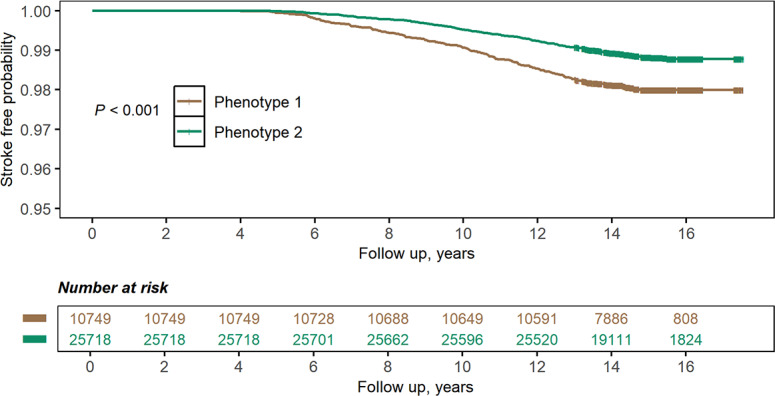
Kaplan-Meier survival curves of stroke-free probability by phenotype.

Kaplan-Meier survival curves revealed that the phenotype 1 group had a higher risk of stroke (*P*<.001; Figure 4).

Regarding stroke subtypes, cardiac function phenotypes were associated with cerebral infarction and other nontraumatic intracranial hemorrhage (all *P*<.05; Table S5 in Multimedia Appendix 1). When mortality was considered as a competing risk, phenotype 2 remained significantly associated with stroke risk (HR 0.578, 95% CI 0.484-0.691; *P*<.001; Figure S8 in Multimedia Appendix 1).

### Supervised ML

**Figure 5.**
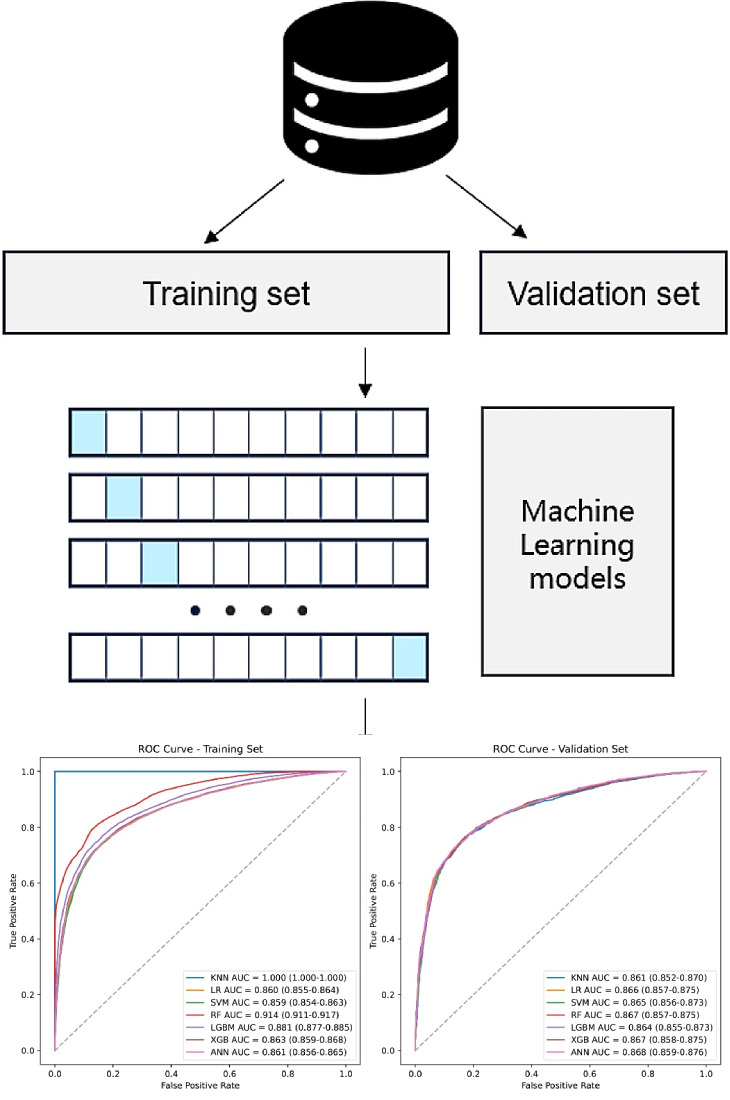
Machine learning for phenotypes. ANN: artificial neural network; AUC: area under the curve; KNN: k-nearest neighbors; LGBM: light gradient boosting machine; LR: logistic regression; RF: random forest; SVM: support vector machine; XGB: extreme gradient boosting (XGBoost).

Basal metabolic rate, sex, testosterone, standing height, weight, forced vital capacity, waist circumference, creatinine, forced expiratory volume in 1 second, and urate were included in the ML models (Table S6 in Multimedia Appendix 1). By integrating the accuracy in the training and validation sets, we selected the random forest model as the optimal model based on its excellent accuracy (area under the curve 0.914, 95% CI 0.911-0.918 for training and 0.867, 95% CI 0.858-0.876 for validation; and accuracy 0.837, 95% CI 0.833-0.841 for training and 0.805, 95% CI 0.804-0.806 for validation) without overfitting the training set (Figure 5). Calibration plots exhibited that the random forest model had good performance in the calibration quality (Brier score 0.111, 95% CI 0.109-0.113 for training and 0.132, 95% CI 0.127-0.137 for validation; Table 3 and Tables S7 and S8 in Multimedia Appendix 1). We drew a feature importance plot to better visualize the contributions of the predictors to the predictive power of the random forest model (Figure S9 in Multimedia Appendix 1).

**Table 3.** Model evaluation and validation.

Models	Training set		Validation set	
	Accuracy (95% CI)	Brier score (95% CI)	Accuracy (95% CI)	Brier score (95% CI)
K-nearest neighbor	1.000 (1.000-1.000)	0.000 (0.000-0.000)	0.800 (0.800-0.800)	0.134 (0.130-0.139)
Logistic regression	0.797 (0.793-0.802)	0.136 (0.134-0.139)	0.802 (0.802-0.802)	0.132 (0.127-0.137)
Support vector machine	0.795 (0.791-0.800)	0.137 (0.135-0.140)	0.802 (0.802-0.802)	0.133 (0.128-0.138)
Random forest	0.837 (0.833-0.841)	0.111 (0.109-0.113)	0.805 (0.804-0.806)	0.132 (0.127-0.137)
Light gradient boosting machine	0.810 (0.805-0.814)	0.126 (0.124-0.128)	0.805 (0.805-0.805)	0.132 (0.128-0.137)
Extreme Gradient Boosting	0.799 (0.795-0.804)	0.134 (0.132-0.137)	0.804 (0.804-0.804)	0.132 (0.127-0.137)
Artificial neural networks	0.797 (0.793-0.802)	0.136 (0.133-0.138)	0.803 (0.803-0.804)	0.132 (0.127-0.136)

After entering the participant information, the local model calculated the corresponding phenotype, and the DeepSeek-R1 model provided an analysis of the participant’s health status along with recommendations for potential clinical application (Figure S10 in Multimedia Appendix 1).

## Discussion

### Principal Findings

In this study, we identified distinct cardiac function phenotypes related to cardiovascular aging using the GTM model based on CMR parameters. These phenotypes were significantly associated with long-term stroke risk and could be reliably predicted by supervised ML models.

CMR is important for evaluating cardiovascular pathologies and for assessing the etiology and prognosis of patients with stroke. A previous study demonstrated that cardiovascular aging is associated with reduced LV volumes and increased LV concentricity on CMR, with notable sex-specific differences in systolic cardiac function [9]. CMR also exhibited high sensitivity in detecting intraventricular thrombi and thrombi within the LA appendage [23]. Additionally, CMR imaging could affect the management of acute stroke by detecting aortic plaques, cardiac structural abnormalities, and intracardiac thrombi in patients with stroke [24,25]. However, CMR metrics represented multidimensional data with complex interrelations, potentially involving collinearity and interaction effects. Using unsupervised ML may improve the ability to reveal the inherent structure of the data and achieve more accurate classification.

GTM incorporated probability distribution functions to refine the self-organizing map algorithms and mitigated their inherent limitations, such as convergence issues, insufficient neighborhood preservation, and the absence of a distinct objective function. Moreover, GTM was capable of visually representing phenotypes through latent spaces [17]. Sattarov et al [26] applied GTM to explore the latent space of Simplified Molecular Input Line Entry System–based autoencoders for generating targeted molecular libraries and visualized the autoencoder latent space on a 2D topographic map. These findings indicated a promising application of GTM in structure-based affinity assessments. However, unsupervised ML faces challenges related to limited interpretability and scalability, making the derived phenotypes and their clinical relevance difficult to translate into real-world medical practice. Therefore, we innovatively applied supervised ML to relearn the phenotype labels obtained from GTM, constructing a random forest model that accurately predicted the 2 distinct phenotypes. Given the high cost and inconvenience of CMR scans for patients with stroke [27], using clinical variables allowed for a reduction in model application requirements while preserving accuracy, making the classification model more suitable for clinical use.

The phenotypes derived from CMR metrics may reflect distinct patterns of cardiac structural and functional remodeling associated with cardiovascular aging. Key variables contributing to phenotype differentiation, including LVMM and concentric geometry, have been linked to vascular aging markers, supporting the connection between cardiovascular aging and cardiac structural changes [28]. LA enlargement and functional impairment are also recognized markers of cardiovascular aging and are related to chronic diastolic dysfunction and atrial fibrillation [29]. In addition, accelerated cardiovascular aging driven by cumulative risk factors such as hypertension, obesity, diabetes, and coronary artery disease may further promote cardiac remodeling, consistent with the differing comorbidity profiles observed between the phenotypes in our study [30]. Notably, the cardiovascular aging–related phenotype in our study was characterized by LV and LA abnormalities indicative of volume or pressure overload and myocardial dysfunction. These alterations have been associated with increased risks of cardioembolic stroke and subclinical cerebral infarction [31,32]. Therefore, the CMR-derived phenotypes may capture different stages of cardiovascular aging and help explain the heterogeneity of cardiac remodeling and its potential contribution to long-term stroke risk.

In our study, supervised ML models identified several clinical parameters as predictors for cardiac function phenotypes. Notably, basal metabolic rate is the energy required to maintain essential functions in a healthy state. Prior research indicated that basal metabolic rate was independently linked to heart failure, atrial fibrillation, and flutter and acted as a risk factor for cardiovascular mortality [33,34]. Furthermore, a pooled analysis of 8 cohorts showed that obstructive and restrictive physiology were associated with incident heart failure with reduced or preserved ejection fraction [35]. Moreover, a distinctive J-shaped curve was noted between BMI and waist circumference and the risk of heart failure [36]. Additionally, renal impairment in chronic heart failure was typically linked to decreased cardiac output and subsequent renal hypoperfusion, and targeting volume overload may improve outcomes in cardiac dysfunction [37]. These findings suggested that the ML model could reflect systemic metabolic, cardiopulmonary, and renal alterations that may explain the clinical characteristics of the identified cardiac function phenotypes.

To the best of our knowledge, this was the first study to use GTM for clustering CMR parameters and to identify cardiovascular aging phenotypes associated with long-term stroke risk. However, there were several limitations to our study. First, the CMR parameters extracted from the UK Biobank were collected according to the cohort’s standardized protocols. Because the CMR examination protocol may differ in other studies, this should be considered when applying our model to other cohorts. Second, while trained on the UK Biobank’s large sample, the model’s applicability to other cohorts may be limited by environmental, genetic, and cultural variations. Third, given the predominantly White and healthy population in the UK Biobank, selection and health biases may be inevitable. The generalizability of our findings to ancestrally diverse individuals will assist in determining whether more appropriate and ethnically relevant decisions are needed.

### Conclusions

In conclusion, we used GTM to identify cardiac function phenotypes based on CMR parameters. The identified phenotypes were significantly associated with stroke risk, with the high-risk phenotype characterized by impaired cardiac function and features related to cardiovascular aging. Furthermore, we relearned phenotype labels using supervised ML models, enabling phenotype prediction from clinical variables. These models may facilitate the identification of individuals at higher risk and support the development of preventive and therapeutic strategies. Further studies are warranted to explore the potential mechanisms linking cardiovascular aging, cardiac phenotypic heterogeneity, and stroke risk.

## Supplementary material

10.2196/77017 Multimedia Appendix 1 Additional methods and results detailing cardiovascular magnetic resonance analysis, generative topographic mapping for cardiac phenotyping, and machine learning frameworks for stroke risk prediction. Includes comprehensive methodology, participant characteristics, and model validation metrics using UK Biobank data.

10.2196/77017 Checklist 1 TRIPOD checklist.

## Abbreviations

API: application programming interface
CMR: cardiovascular magnetic resonance
GTM: generative topographic mapping
HR: hazard ratio
LA: left atrial
LAEF: left atrial ejection fraction
LAMV: left atrial maximum volume
LV: left ventricular
LVEDV: left ventricular end-diastolic volume
LVEF: left ventricular ejection fraction
LVESV: left ventricular end-systolic volume
LVGLS: left ventricular global longitudinal strain
LVMM: left ventricular myocardial mass
LVSV: left ventricular stroke volume
ML: machine learning
TRIPOD: transparent reporting of a multivariable prediction model for individual prognosis or diagnosis
