# Supplementary material for: Machine Learning–Derived Cardiovascular Aging Phenotypes From Cardiac Function and Stroke Risk in the UK Biobank: Cohort Study
Source: JMIR Aging. 2026 Apr 27;9:e77017. doi: 10.2196/77017 (PMC13120752; doi:10.2196/77017)
Supplement: Multimedia Appendix 1 [file aging-v9-e77017-s001.docx]

**Supplementary materials**

**Supplementary Methods**

**CMR Image Acquisition and Analysis**

The UK Biobank imaging study, initiated in 2015, focused on a random subset of 100,000 original participants undergoing comprehensive cardiovascular magnetic resonance (CMR) imaging assessments[1]. CMR imaging was performed with 1.5 T scanners (MAGNETOM Aera, Syngo Platform VD13A, Siemens Healthcare, Erlangen, Germany)[2]. The assessment of cardiac function assessment included 3 long-axis cine images and a comprehensive short-axis stack on the left and right ventricles. Images were acquired with a balanced steady-state free precession sequence at a rate of one slice per breath-hold. CMR indices were analyzed using a fully automated, quality-controlled pipeline that was developed and validated in previous studies[3,4]. We considered the following CMR measures in our study: left ventricular (LV) stroke volume (LVSV), LV myocardial mass (LVMM), LV end diastolic volume (LVEDV), LV end systolic volume (LVSDV), LV ejection fraction (LVEF), LV longitudinal strain global (LVGLS), left atrial (LA) maximum volume (LAMV), and LA ejection fraction (LAEF).

**GTM Modeling and Phenotype Generating**

Generative Topographic Mapping (GTM) is a probabilistic model used for dimensionality reduction and data visualization, that functions as a principled alternative to the widely used Self Organizing Map algorithm (SOM)[5,6]. GTM takes advantages in uncertainty quantification, robustness to noise, and the identification of hidden subgroups. Compared to heuristic methods like t-SNE and UMAP, GTM is particularly effective for interpreting complex data structure, visualizing clusters, detecting patterns, and providing a more reliable stratification of high-dimensional datasets. The core principle of the GTM model lies in mapping high-dimensional data onto a lower-dimensional latent space (typically 2D) to visualize the underlying structure of the data. GTM assumes that observed data are generated from a lower-dimensional latent space through a nonlinear mapping governed by a set of probability density functions (e.g., radial basis functions). GTM uses a Gaussian mixture model to picture the distribution in the latent space and applies the Expectation-Maximization (EM) algorithm to optimize the parameters in the Gaussian mixture model, which ensures the mapping process is adjusted iteratively to best represent the underlying data structure in the latent space. After optimization with the EM algorithm, GTM employs a soft assignment method to estimate the probability of each participant belonging to various clusters. The cluster with the highest probability determines the final assignment of the participants. As a result, participants with similar characteristics are grouped into the same cluster, reducing the likelihood of including outliers or dissimilar members. The original high-dimensional data can then be visualized in a 2D latent space. The latent space clearly illustrates the distribution of participants across various latent space nodes, providing a clear visualization of how participants are grouped based on shared characteristics or patterns in the original dataset.

To get a robust and representative dataset for modeling, we excluded variables with more than 25% missing values and participants with over 30% missing data. Variables with positively skewed distributions were log-transformed to approximate a Gaussian distribution. Remaining missing data were imputed using “IterativeImputer” function in the “Scikit-Learn” package. After preprocessing the dataset, we utilized the "ugtm" Python package for GTM modeling. Although hyperparameter tuning is generally less critical for GTM, we applied the cross-validation method to minimize overfitting and improve generalization. We conducted a grid search within a predefined parameter space to optimize the hyperparameters of the GTM model. The hyperparameters included the number of latent grid nodes, the number of Gaussian centers, the number of radial basis functions (RBFs), and the penalization term. Each combination of hyperparameters was evaluated using 10-fold cross-validation to achieve the optimal negative log-likelihood of the test fold of dataset. The hyperparameter tuning results identified an optimal parameter set comprising the following: a number of latent grid nodes equal to 16, 4 RBFs, an RBF function width factor of 0.2, and a regularization term of 0.001.

After training the GTM model, the dataset was visualized as a 2D heatmap. The heatmap represented the data distribution in the observed data space, with cluster centers denoted by yellow-blue scheme circles. The intensity of the colors indicates the density of participants in each cluster, with darker colors representing higher densities. The cardiac function parameters illustrated the impact of cardiac function on each latent space node in the GTM model. Darker colors in the latent space nodes corresponded to higher values of the modeling variables. To provide a more comprehensive understanding of the latent space, investigatory variables were superimposed onto the latent space nodes. These investigatory heatmaps aligned point-for-point with the latent space, offering deeper insights into the characteristics of the clusters derived through the GTM modeling.

To generate the cardiac function phenotypes, we calculated the distance between the latent space nodes using Euclidean distances and applied agglomerative hierarchical clustering on the latent space nodes using Ward's minimum variance method[7]. The resulting cluster assignments were subsequently mapped back onto the latent space, revealing the delineation of phenotypes, which aggregated the latent space nodes and facilitated the identification of meaningful patterns that correspond to distinct cardiac function phenotypes.

**Supervised Machine Learning Frameworks**

After labeling participants as different phenotypes using the GTM approach, we trained multiple supervised machine learning algorithms to predict the high- and low-risk phenotypes for cardiac function. The supervised machine learning process included the following steps: automated variable selection using variable importance, model training using machine learning algorithms, hyperparameters tuning with cross validation method and model validation to assess the accuracy and the calibration.

We initially included investigatory variables such as demographic characteristics, physical measurements, family history, medication use, and blood assay data from baseline visits. The dataset was preprocessed by handling missing values, outliers, and duplicates. Variables with more than 25% missing values and those with *P* value ≥ .1 in univariable analyses were excluded as non-informative. Remaining missing data were imputed, numeric variables were normalized, and multi-level categorical variables were one-hot encoded. Attribute importance scores were calculated using the Information Gain method implemented in the FSelector package in R. Variables were ranked according to their information gain values, and the top 10 features were selected for training the ML models.

We implemented several important machine learning models, including K-nearest neighbors, logistic regression, support vector machines, random forests, light gradient boosting machine, extreme gradient boosting, and artificial neural networks, to predict cardiac function phenotypes. The hyperparameters for each model are detailed in **Table S3** in **Multimedia Appendix 1**. We split the data into training and validation sets in an 8:2 ratio. To enhance model reliability and mitigate overfitting or underfitting due to random train-test splits, we performed 10-fold cross-validation on the training set, averaging accuracy scores across folds for a comprehensive evaluation[8]. Hyperparameter optimization was conducted using grid search and Bayesian hyperparameter search techniques.

After finding the optimal hyperparameters for each model, the models were refitted using the entire training set and subsequently validated on the validation set to assess their performance. Model performance was evaluated using multiple metrics, including accuracy, precision, sensitivity, specificity, F1 score, and Brier score with the 1000 bootstrapping method. Discriminatory ability was evaluated using the receiver operating characteristic (ROC) curve, with the area under the curve (AUC) quantifying its accuracy. Calibration performance was assessed using the calibration curve, with the Brier score representing the calibration quality. We selected the best-performing method as the final algorithm for developing a cardiac function phenotype prediction model.

Using the best-performing machine learning model stored in the local computer, we developed a web application with flask package in Python. After entering the individualized metrics in the website, we calculated the predicted probabilities for phenotype classification. Then, we accessed the Deepseek API in the flask framework and sent the prompt to the Deepseek-R1 model by combining the predictive probabilities with the individualized metrics. We defined the role of Deepseek as an expertise in clinical medicine and asked Deepseek for a detailed analysis of the health status of the participant and get professional recommendation.

**Figure S1. Visualization of Stroke Diagnoses of the Participants by Latent Spaces Based on ICD-10 Codes.**


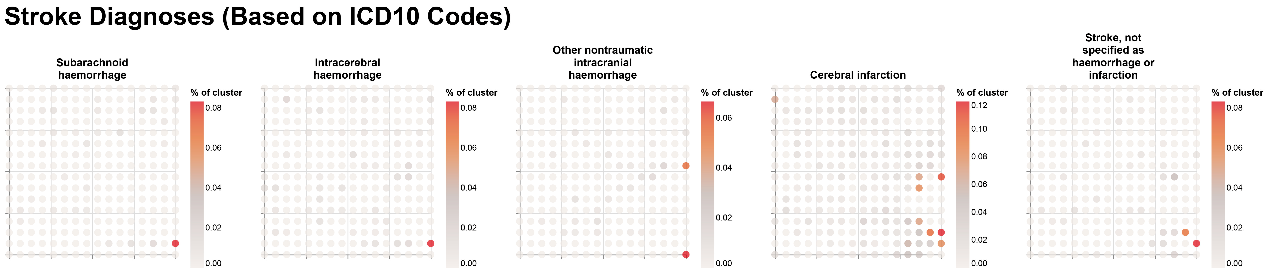


**Figure S2. Visualization of Demographic Characteristics of the Participants by Latent Spaces.**


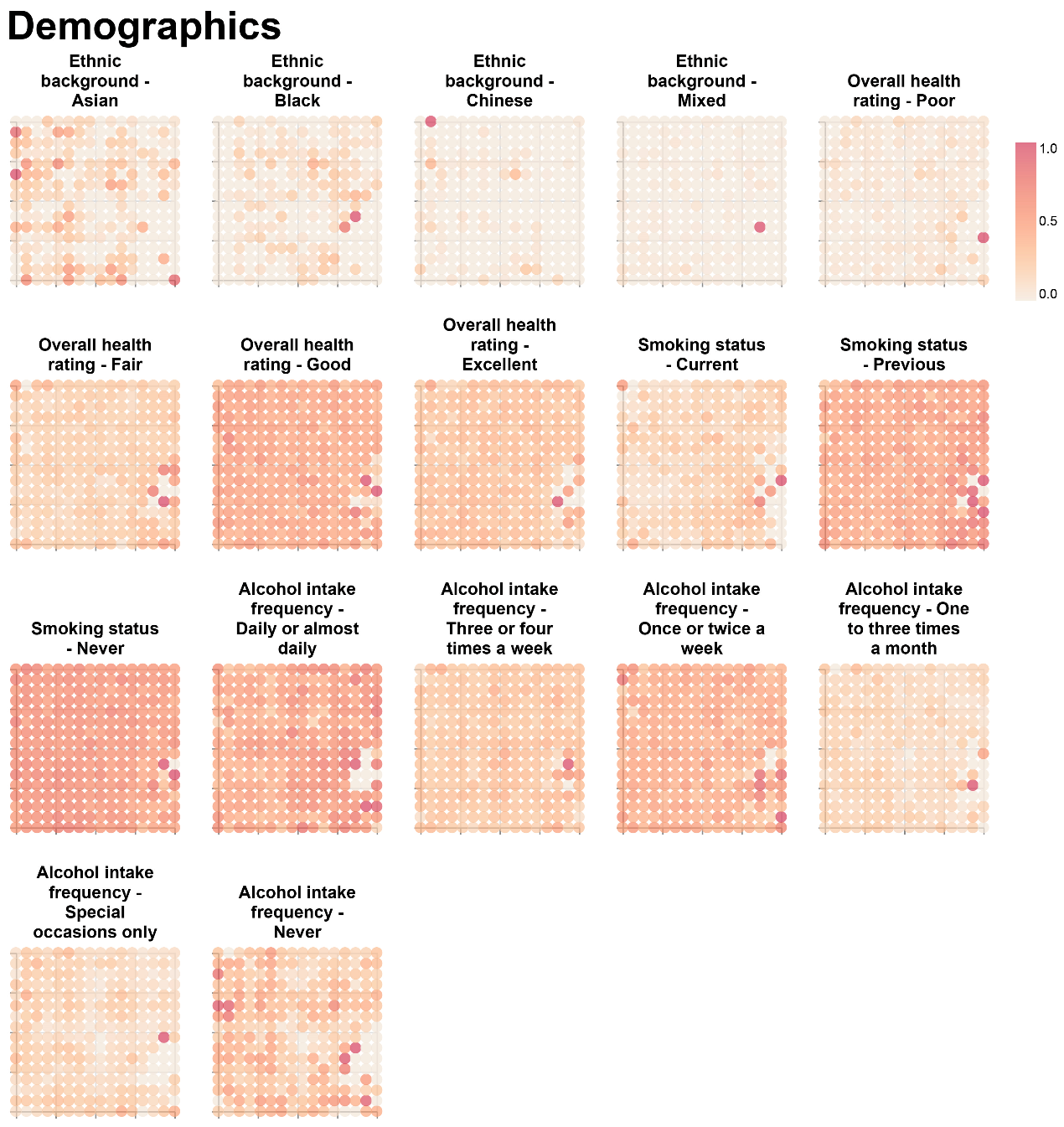


**Figure S3. Visualization of Physical Measurements of the Participants by Latent Spaces.**


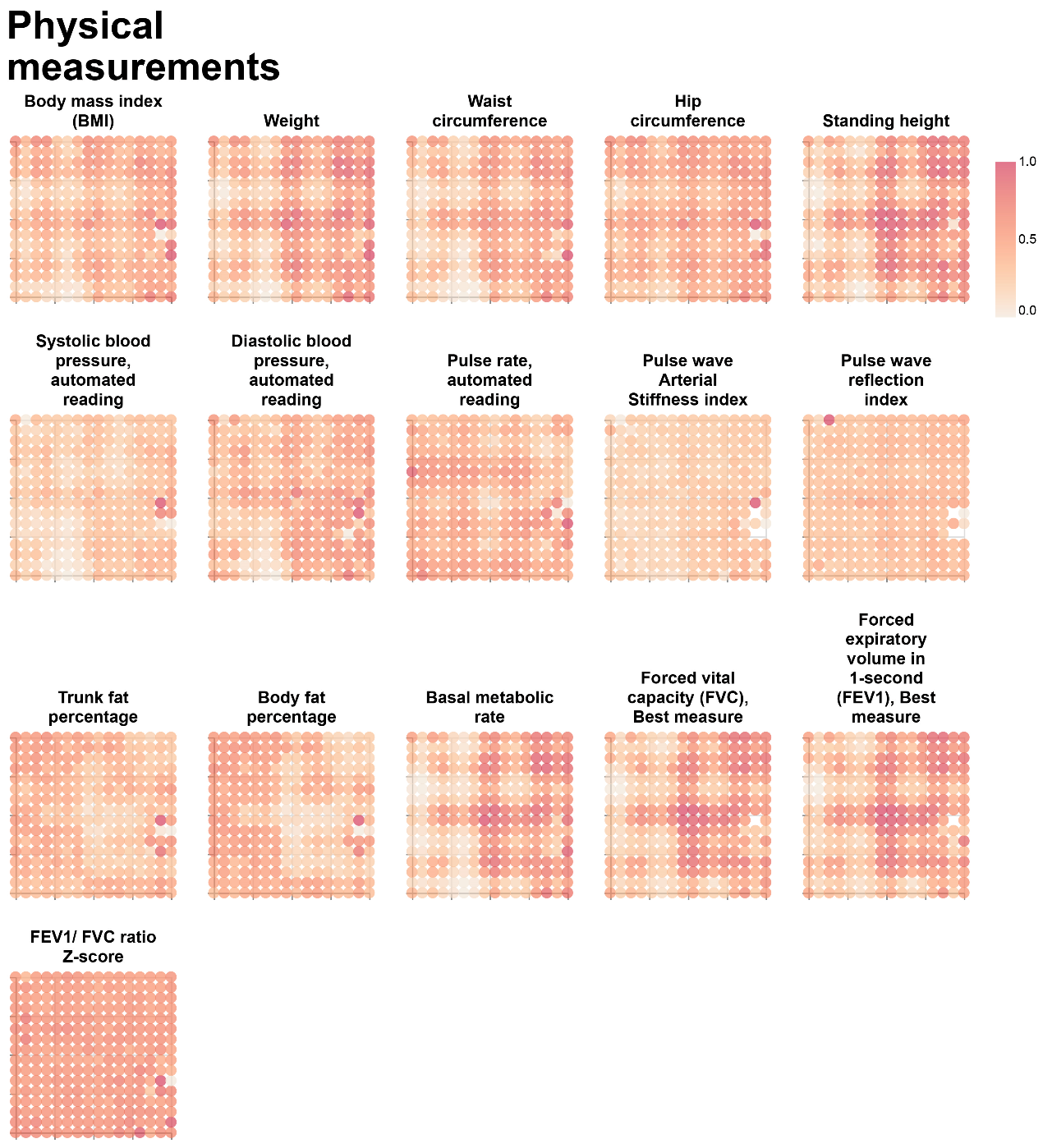


**Figure S4. Visualization of Biological Samples of the Participants by Latent Spaces.**


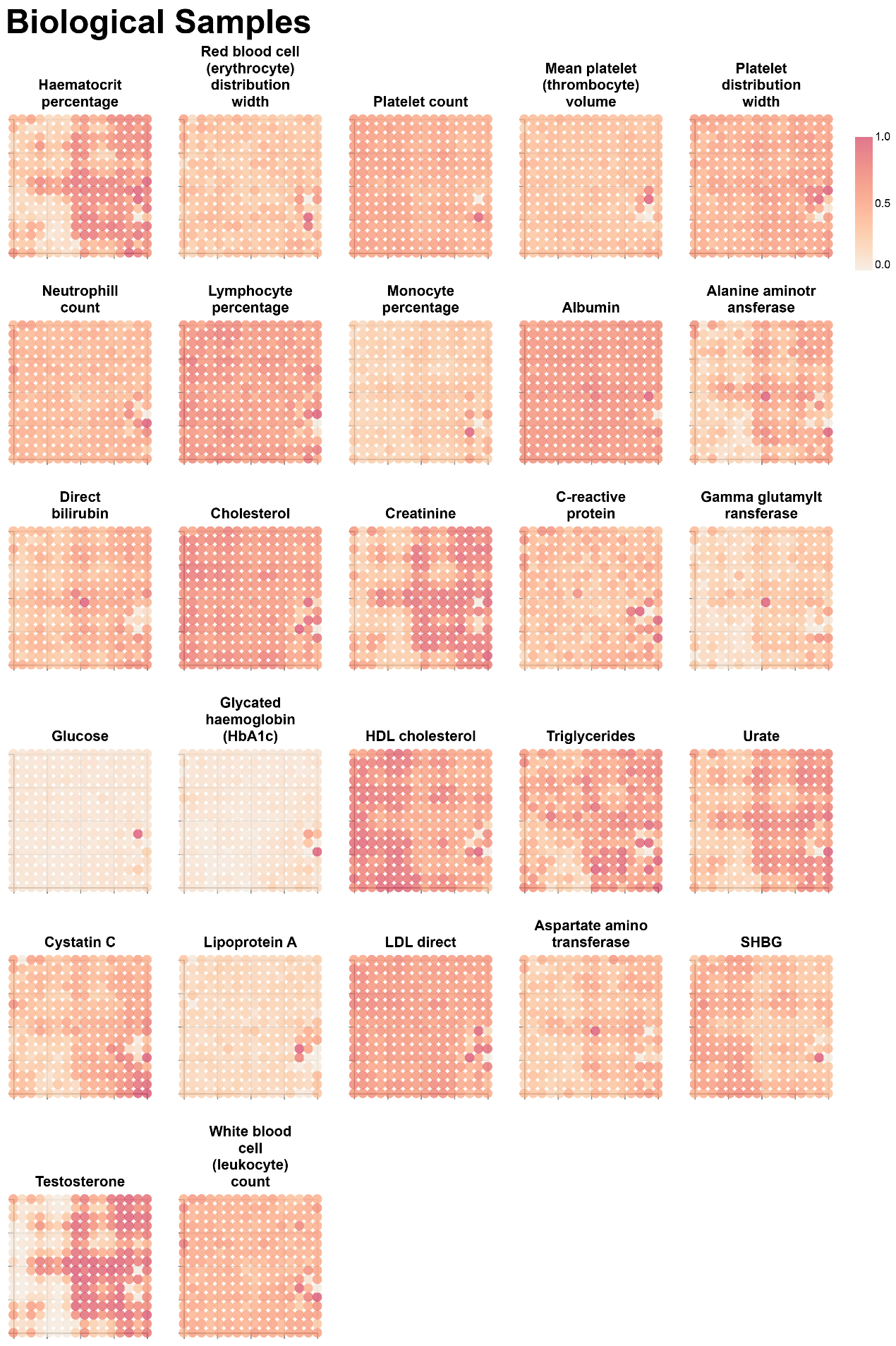


**Figure S5. Visualization of Family history of the Participants by Latent Spaces.**


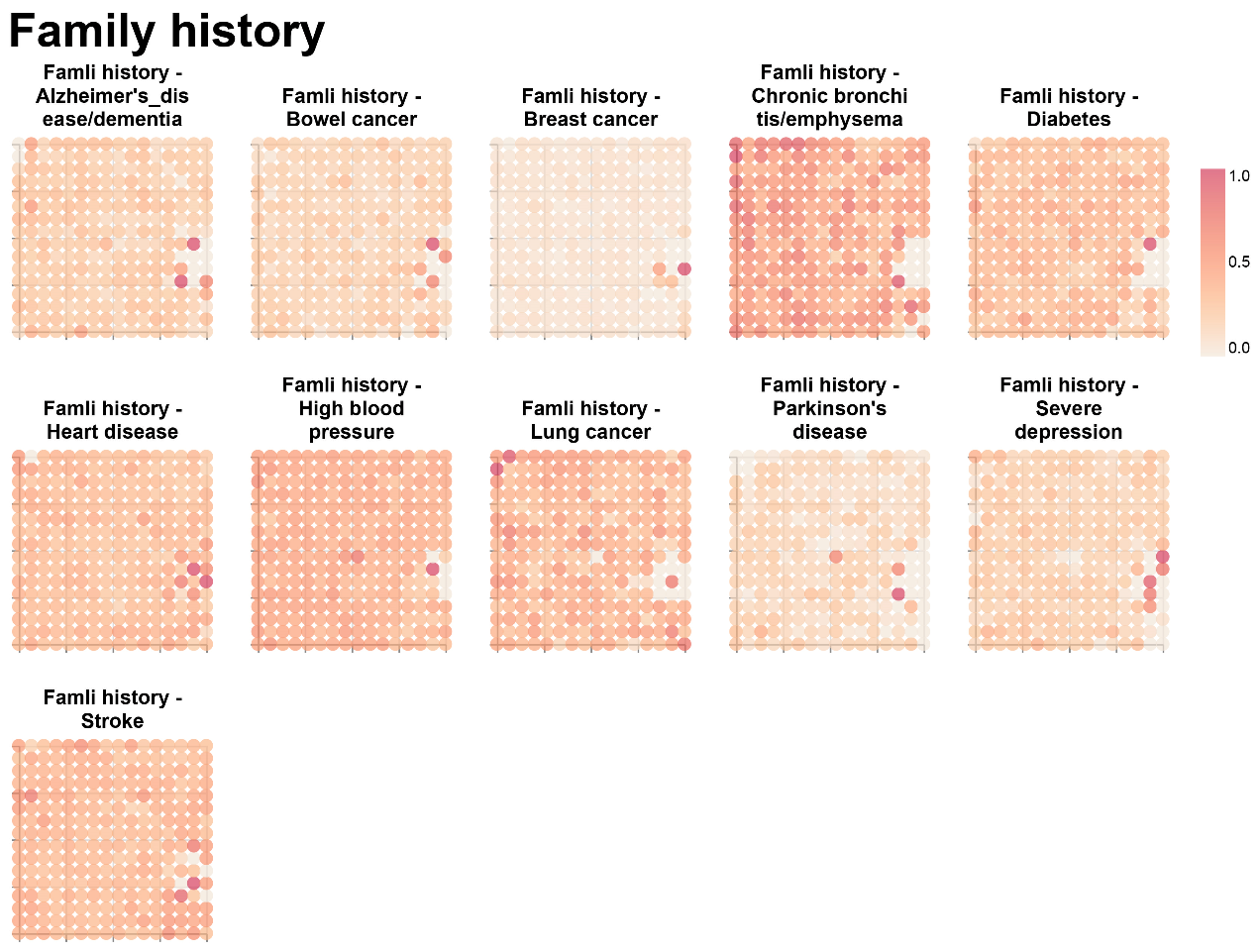


**Figure S6. Visualization of Comorbidities of the Participants by Latent Spaces.**


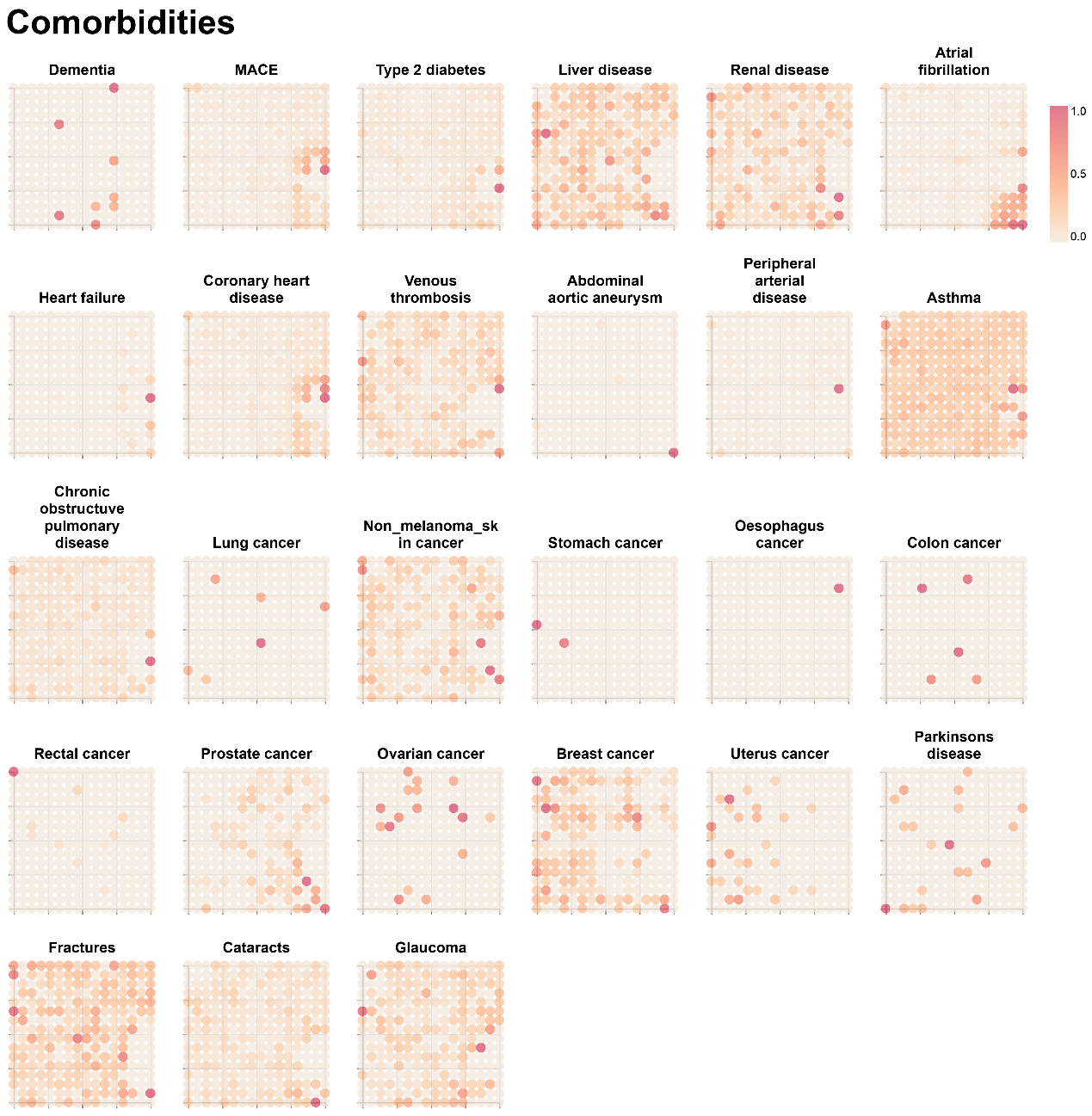


**Figure S7. Visualization of Medication of the Participants by Latent Spaces.**


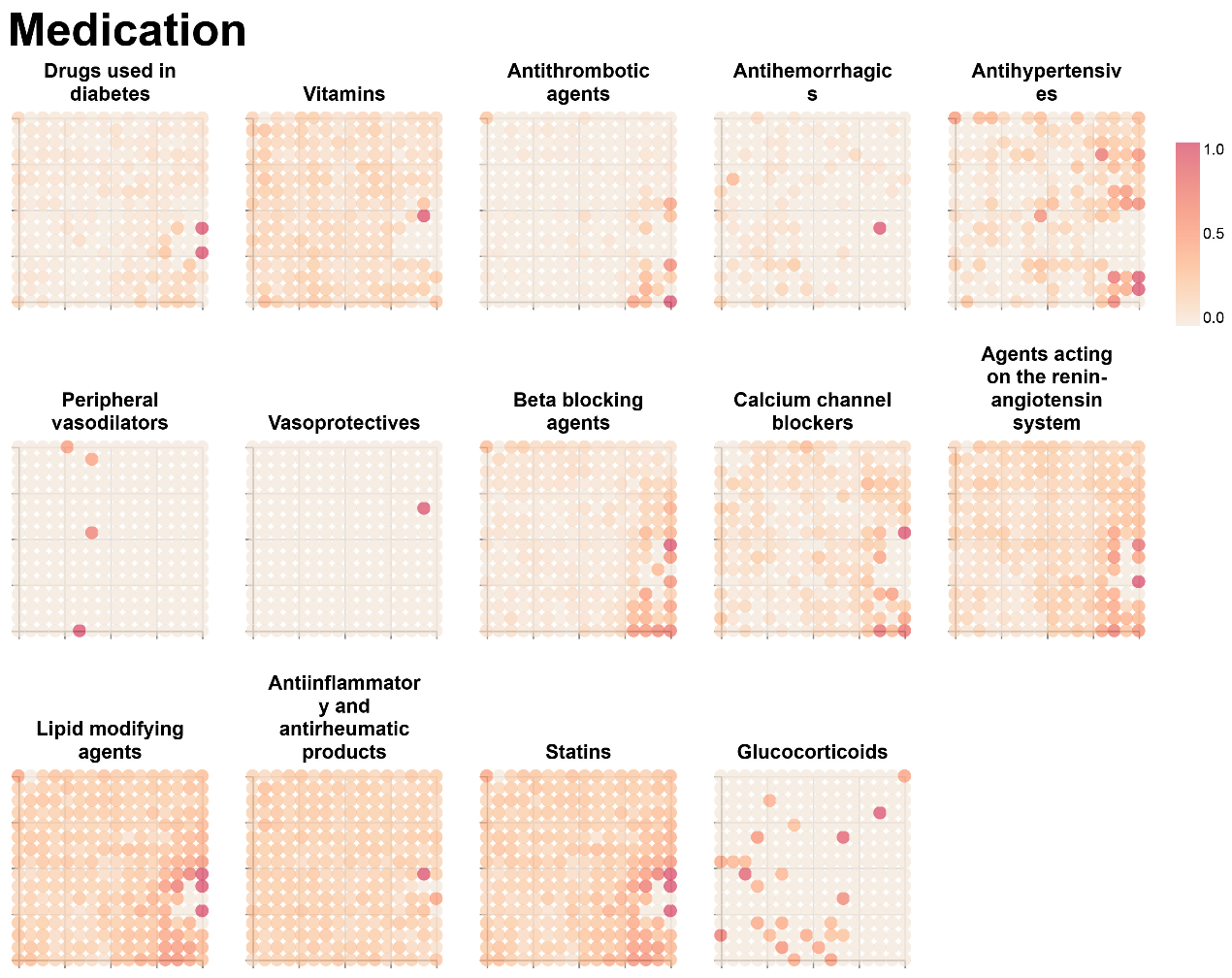


**Figure S8. Cumulative Incidence Curves for the Association of Phenotypes of Cardiac Function and Competing Risks of Stroke and Death. The purple and orange areas represented the cumulative incidence of stroke risk and death, respectively. The hazard ratio for the association between cardiac function phenotypes and long-term risk of stroke was 0.578 (95% confidence interval, 0.484-0.691; *P* < .001), with mortality considered as a competing risk using the Fine and Gray method.**


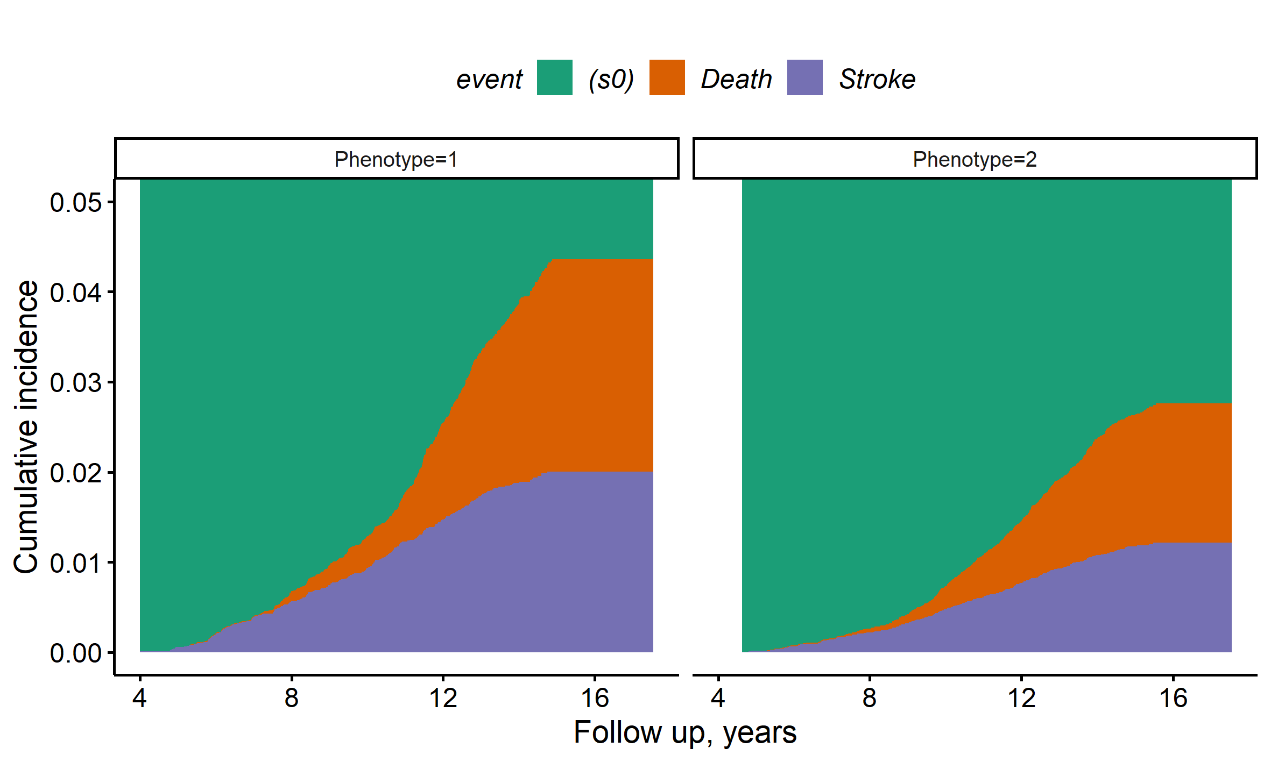


**Figure S9. Performance of machine learning models for predicting phenotypes of cardiac function in the training and validation cohorts. The performance of the machine learning models was depicted through the ROC curves (A and B) and calibration curves (C and D), which assessed the models' discrimination and calibration ability, respectively, in both the training and validation sets. RF was selected as the optimal model, and the feature importance was illustrated in (E). The feature importance plot displayed predictors on the y-axis and their importance scores on the x-axis by evaluating how much each feature reduces impurity when used to split the data at a node across all trees. ANN, artificial neural networks; AUC, area under the curve; FEV1, forced expiratory volume in 1-second; FVC, forced vital capacity; KNN, K-nearest neighbor; LGBM, light gradient boosting machine; LR, logistic regression; RF, random forest; ROC, receiver operating characteristic; SVM, support vector machine; XGB, eXtreme Gradient Boosting.**


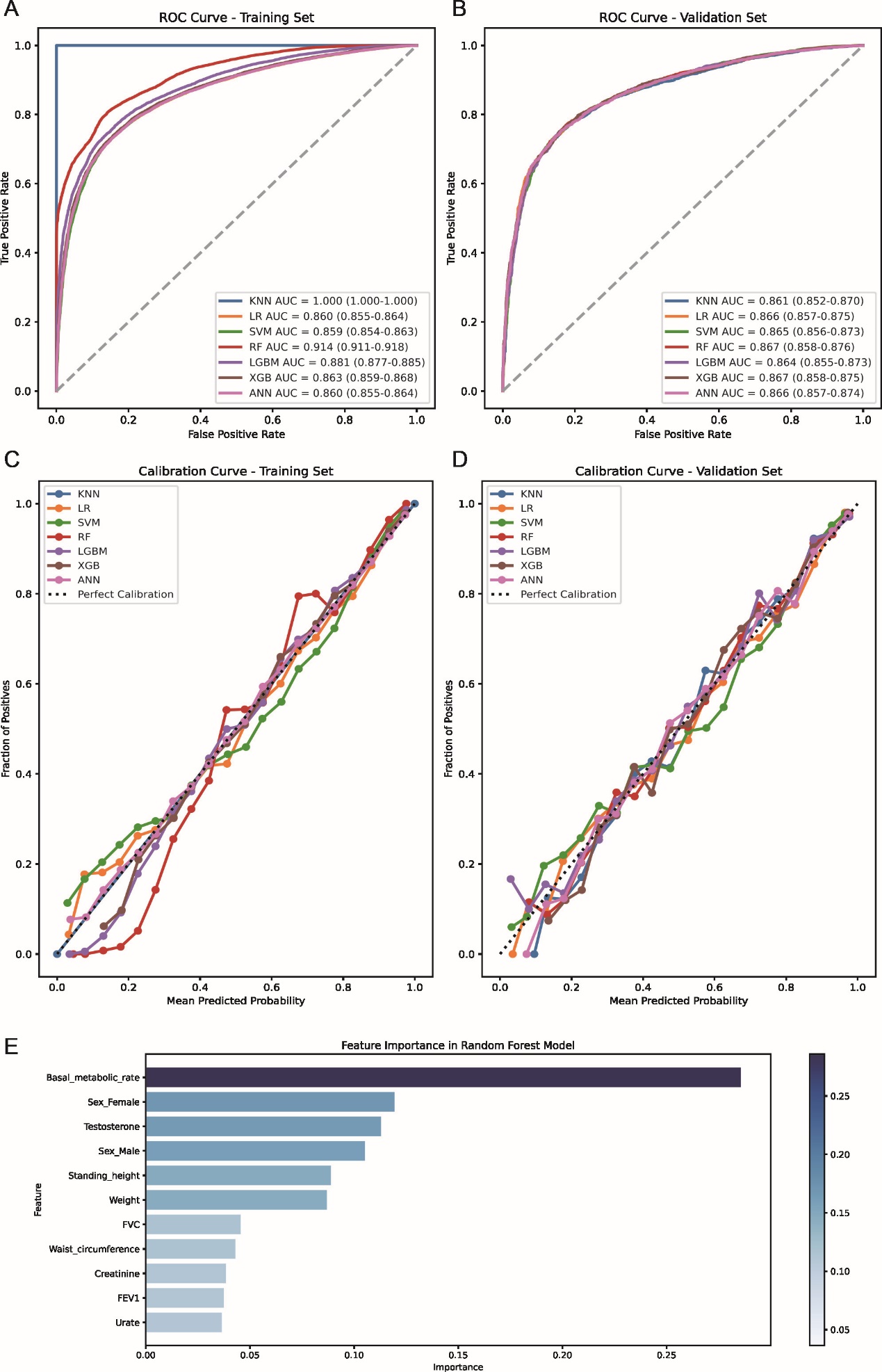


**Figure S10. The Health Consultation Webpage Featuring Machine Learning and DeepSeek Models. The model can be downloaded from GitHub at https://github.com/qianwg/API-for-CMR# and deployed locally using the Flask framework with the personal DeepSeek-R1 API key.**


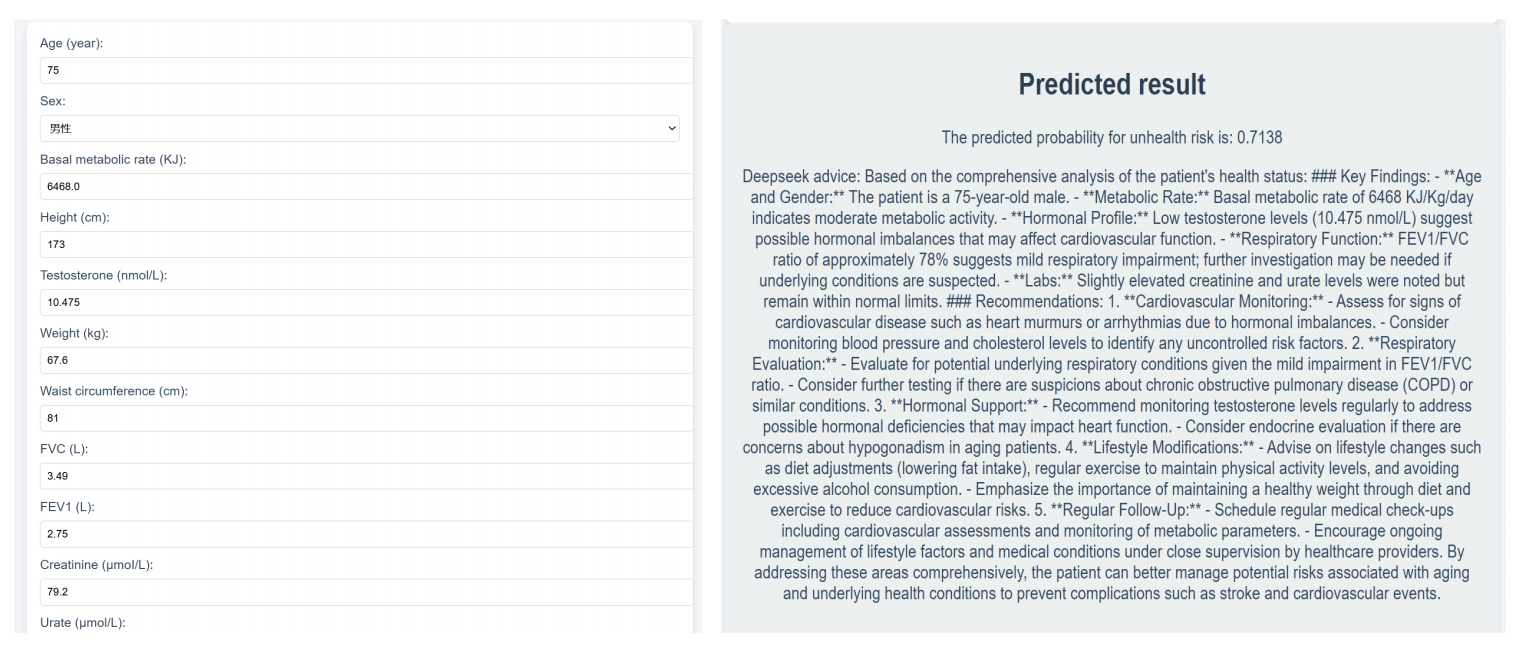


**Table S1. Codes List for Stroke in the UK Biobank.**

| **Code Type** | **Code** | **Code Description** |
| --- | --- | --- |
| Self-reported medical conditions | Field 20002 Code 1081 | Stroke |
|  | Field 20002 Code 1086 | Subarachnoid hemorrhage |
|  | Field 20002 Code 1491 | Brain hemorrhage |
|  | Field 20002 Code 1583 | Ischemic stroke |
| ICD-9 | 430.X | Subarachnoid hemorrhage |
|  | 431.X | Intracerebral hemorrhage |
|  | 434.X | Occlusion of cerebral arteries |
|  | 436.X | Acute, but ill-defined, cerebrovascular disease |
| ICD-10 | I600 | Subarachnoid hemorrhage |
|  | I601 | Subarachnoid hemorrhage |
|  | I602 | Subarachnoid hemorrhage |
|  | I603 | Subarachnoid hemorrhage |
|  | I604 | Subarachnoid hemorrhage |
|  | I605 | Subarachnoid hemorrhage |
|  | I606 | Subarachnoid hemorrhage |
|  | I607 | Subarachnoid hemorrhage |
|  | I608 | Subarachnoid hemorrhage |
|  | I609 | Subarachnoid hemorrhage |
|  | I610 | Intracerebral hemorrhage |
|  | I611 | Intracerebral hemorrhage |
|  | I612 | Intracerebral hemorrhage |
|  | I613 | Intracerebral hemorrhage |
|  | I614 | Intracerebral hemorrhage |
|  | I615 | Intracerebral hemorrhage |
|  | I616 | Intracerebral hemorrhage |
|  | I618 | Intracerebral hemorrhage |
|  | I619 | Intracerebral hemorrhage |
|  | I620 | Other nontraumatic intracranial hemorrhage |
|  | I621 | Other nontraumatic intracranial hemorrhage |
|  | I629 | Other nontraumatic intracranial hemorrhage |
|  | I630 | Cerebral infarction |
|  | I631 | Cerebral infarction |
|  | I632 | Cerebral infarction |
|  | I633 | Cerebral infarction |
|  | I634 | Cerebral infarction |
|  | I635 | Cerebral infarction |
|  | I636 | Cerebral infarction |
|  | I638 | Cerebral infarction |
|  | I639 | Cerebral infarction |
|  | I64 | Stroke, not specified as hemorrhage or infarction |

**Table S2. Code List for Candidate Covariates in the UK Biobank.**

| **Category** | **Field IDs and codes** | **Covariates** |
| --- | --- | --- |
| Demographic | 21022 | Age at recruitment |
|  | 31 | Gender |
|  | 21000 | Ethnic background |
|  | 22189 | Townsend deprivation index at recruitment |
|  | 2178 | Overall health rating |
|  | 20116 | Smoking status |
|  | 1558 | Alcohol intake frequency |
| Physical Measurements | 21001 | Body mass index |
|  | 21002 | Weight |
|  | 48 | Waist circumference |
|  | 49 | Hip circumference |
|  | 50 | Standing height |
|  | 4080 | Systolic blood pressure |
|  | 4079 | Diastolic blood pressure |
|  | 102 | Pulse rate |
|  | 21021 | Arterial Stiffness index |
|  | 4195 | Reflection index |
|  | 23127 | Trunk fat percentage |
|  | 23099 | Body fat percentage |
|  | 23105 | Basal metabolic rate |
|  | 20151 | Forced vital capacity |
|  | 20150 | Forced expiratory volume in 1-second |
|  | 20258 | Forced expiratory volume in 1-second / Forced vital capacity ratio |
| Family history | 20107 | Illnesses of father |
|  | 20110 | Illnesses of mother |
| Medication | Field 20003 Code C02 | Drugs used in diabetes |
|  | Field 20003 Code A11 | Vitamins |
|  | Field 20003 Code B01 | Antithrombotic agents |
|  | Field 20003 Code B02 | Antihemorrhagics |
|  | Field 20003 Code C02 | Antihypertensives |
|  | Field 20003 Code C04 | Peripheral vasodilators |
|  | Field 20003 Code C05 | Vasoprotectives |
|  | Field 20003 Code C07 | Beta blocking agents |
|  | Field 20003 Code C08 | Calcium channel blockers |
|  | Field 20003 Code C09 | Agents acting on the renin-angiotensin system |
|  | Field 20003 Code C10 | Lipid modifying agents |
|  | Field 20003 Code M01 | Anti-inflammatory and antirheumatic products |
|  | Field 20003 Code 10A | Statins |
|  | Field 20003 Code 10B | Statins |
|  | Field 20003 Code H02 | Glucocorticoids |
| Blood Assays | 30000 | White blood cell count |
|  | 30030 | Hematocrit percentage |
|  | 30070 | Red blood cell distribution width |
|  | 30080 | Platelet count |
|  | 30100 | Mean platelet volume |
|  | 30110 | Platelet distribution width |
|  | 30140 | Neutrophil count |
|  | 30180 | Lymphocyte percentage |
|  | 30190 | Monocyte percentage |
|  | 30600 | Albumin |
|  | 30620 | Alanine aminotransferase |
|  | 30650 | Aspartate aminotransferase |
|  | 30660 | Direct bilirubin |
|  | 30690 | Cholesterol |
|  | 30700 | Creatinine |
|  | 30710 | C-reactive protein |
|  | 30720 | Cystatin C |
|  | 30730 | Gamma glutamyltransferase |
|  | 30740 | Glucose |
|  | 30750 | Glycated hemoglobin |
|  | 30760 | High-density lipoprotein cholesterol |
|  | 30780 | Ligh-density lipoprotein direct |
|  | 30790 | Lipoprotein A |
|  | 30830 | Sex hormone-binding globulin |
|  | 30850 | Testosterone |
|  | 30870 | Triglycerides |
|  | 30880 | Urate |
| Cardiac Function | 24102 | Left ventricular stroke volume |
|  | 24105 | Left ventricular myocardial mass |
|  | 24100 | Left ventricular end diastolic volume |
|  | 24101 | Left ventricular end systolic volume |
|  | 24103 | Left ventricular ejection fraction |
|  | 24181 | Left ventricular longitudinal strain global |
|  | 24110 | Left atrial maximum volume |
|  | 24113 | Left atrial ejection fraction |
| Comorbidity | F00 | Dementia |
|  | F01 | Dementia |
|  | F02 | Dementia |
|  | F03 | Dementia |
|  | G30 | Dementia |
|  | G31 | Dementia |
|  | G45 | Major adverse cardiac events |
|  | I21 | Major adverse cardiac events |
|  | I22 | Major adverse cardiac events |
|  | I23 | Major adverse cardiac events |
|  | I24 | Major adverse cardiac events |
|  | I25 | Major adverse cardiac events |
|  | I63 | Major adverse cardiac events |
|  | I64 | Major adverse cardiac events |
|  | E10 | Type 2 diabetes |
|  | E11 | Type 2 diabetes |
|  | E12 | Type 2 diabetes |
|  | E13 | Type 2 diabetes |
|  | E14 | Type 2 diabetes |
|  | B15 | Liver disease |
|  | B16 | Liver disease |
|  | B17 | Liver disease |
|  | B18 | Liver disease |
|  | B19 | Liver disease |
|  | C22 | Liver disease |
|  | E83 | Liver disease |
|  | E88 | Liver disease |
|  | I85 | Liver disease |
|  | K70 | Liver disease |
|  | K72 | Liver disease |
|  | K73 | Liver disease |
|  | K74 | Liver disease |
|  | K75 | Liver disease |
|  | K76 | Liver disease |
|  | R18 | Liver disease |
|  | Z94 | Liver disease |
|  | N00 | Renal disease |
|  | N01 | Renal disease |
|  | N02 | Renal disease |
|  | N03 | Renal disease |
|  | N04 | Renal disease |
|  | N05 | Renal disease |
|  | N06 | Renal disease |
|  | N07 | Renal disease |
|  | N08 | Renal disease |
|  | N09 | Renal disease |
|  | N10 | Renal disease |
|  | N11 | Renal disease |
|  | N12 | Renal disease |
|  | N13 | Renal disease |
|  | N14 | Renal disease |
|  | N15 | Renal disease |
|  | N16 | Renal disease |
|  | N17 | Renal disease |
|  | N18 | Renal disease |
|  | N19 | Renal disease |
|  | N25 | Renal disease |
|  | N26 | Renal disease |
|  | N27 | Renal disease |
|  | N28 | Renal disease |
|  | N29 | Renal disease |
|  | I48 | Atrial fibrillation |
|  | I50 | Heart failure |
|  | I20 | Coronary heart disease |
|  | I21 | Coronary heart disease |
|  | I22 | Coronary heart disease |
|  | I23 | Coronary heart disease |
|  | I24 | Coronary heart disease |
|  | I25 | Coronary heart disease |
|  | I80 | Venous thrombosis |
|  | I81 | Venous thrombosis |
|  | I82 | Venous thrombosis |
|  | I71 | Abdominal aortic aneurysm |
|  | I70 | Peripheral arterial disease |
|  | I71 | Peripheral arterial disease |
|  | I72 | Peripheral arterial disease |
|  | I73 | Peripheral arterial disease |
|  | I74 | Peripheral arterial disease |
|  | I75 | Peripheral arterial disease |
|  | I76 | Peripheral arterial disease |
|  | I77 | Peripheral arterial disease |
|  | I78 | Peripheral arterial disease |
|  | I79 | Peripheral arterial disease |
|  | J45 | Asthma |
|  | J46 | Asthma |
|  | J40 | Chronic obstructive pulmonary disease |
|  | J41 | Chronic obstructive pulmonary disease |
|  | J42 | Chronic obstructive pulmonary disease |
|  | J43 | Chronic obstructive pulmonary disease |
|  | J44 | Chronic obstructive pulmonary disease |
|  | J47 | Chronic obstructive pulmonary disease |
|  | C33 | Lung cancer |
|  | C34 | Lung cancer |
|  | C44 | Non melanoma skin cancer |
|  | C16 | Stomach cancer |
|  | C15 | Esophagus cancer |
|  | C18 | Colon cancer |
|  | C19 | Rectal cancer |
|  | C20 | Rectal cancer |
|  | C61 | Prostate cancer |
|  | C56 | Ovarian cancer |
|  | C57 | Ovarian cancer |
|  | C50 | Breast cancer |
|  | C54 | Uterus cancer |
|  | G20 | Parkinsons disease |
|  | G21 | Parkinsons disease |
|  | G22 | Parkinsons disease |
|  | S02 | Fractures |
|  | S12 | Fractures |
|  | S22 | Fractures |
|  | S32 | Fractures |
|  | S42 | Fractures |
|  | S52 | Fractures |
|  | S62 | Fractures |
|  | S72 | Fractures |
|  | S82 | Fractures |
|  | S92 | Fractures |
|  | T02 | Fractures |
|  | T08 | Fractures |
|  | T10 | Fractures |
|  | H25 | Cataracts |
|  | H26 | Cataracts |
|  | H40 | Glaucoma |

**Table S3. Hyperparameter Tunning Grids for Machine Learning Models.**

| **Models** | **Hyperparameters** | **Range** | **Optimal parameters** |
| --- | --- | --- | --- |
| K-nearest neighbor | n_neighbors | [10-100] | 100 |
|  | weights | {uniform, distance} | distance |
|  | algorithm | {auto, ball_tree, kd_tree, brute} | auto |
| Logistic regression | solver | {liblinear, lbfgs, saga} | lbfgs |
|  | penalty | {l1, l2, elasticnet, none} | l2 |
|  | C | {0.001, 0.01, 0.05, 0.1, 0.5, 1} | 0.01 |
| Support vector machine | kernel | {rbf, sigmoid} | sigmoid |
|  | C | {0.001, 0.01, 0.1, 1, 10, 100, 1000} | 10 |
|  | gamma | {scale, auto, 0.001, 0.01, 0.1} | 0.001 |
| Random forest | n_estimators | {100, 200, 300, 400, 500, 600, 700, 800, 900, 1000} | 900 |
|  | criterion | {gini, entropy} | entropy |
|  | max_depth | {3, 5, 7, 9, 11, 13, 15} | 13 |
|  | min_samples_leaf | {3, 5, 7, 9, 11, 13, 15} | 13 |
|  | min_samples_split | {3, 5, 7, 9, 11, 13, 15} | 9 |
|  | max_features | {none, sqrt, log2} | sqrt |
| Light gradient boosting machine | n_estimators | [100, 1000] | 668 |
|  | max_depth | [3, 30] | 3 |
|  | subsample | [0.7, 1] | 0.7 |
|  | colsample_bytree | [0.7, 1] | 0.731108000463631 |
|  | learning_rate | [1e-5, 1e-1] | 0.05341439749195817 |
|  | num_leaves | [10, 100] | 10 |
| eXtreme Gradient Boosting | n_estimators | [100, 1000] | 100 |
|  | max_depth | [3, 15] | 3 |
|  | min_child_weight | [3, 15] | 5 |
|  | subsample | [0.7, 1] | 0.7 |
|  | eta | [1e-5, 1e-1] | 0.045644362194042513 |
| Artificial neural networks | num_layers | [1, 5] | 1 |
|  | units | {3, 5, 7, 10} | 10, 3, 7, 10, 3 |
|  | dropout_rate | {0, 0.05, 0.1, 0.15, 0.2, 0.25, 0.3, 0.35, 0.4, 0.45, 0.5} | 0 |
|  | optimizer | {Adam, Adamax, SGD, RMSprop} | Adam |
|  | learning_rate | [1e-5, 1e-1] | 0.00023708664785748507 |
|  | batch_size | [128, 256, 512, 1024] | 512 |

**Table S4. Comorbidity, Physical examination, Family History, Medications and Blood Assays of the Participants.**

|  | **Total** | **Phenotype 1** | **Phenotype 2** |  |  |  |
| --- | --- | --- | --- | --- | --- | --- |
| **Characteristics** | **N = 36467** | **N = 10749** | **N = 25718** | **Statistics** | ***P* value** | ***P* value^a^** |
| Comorbidity, n (%) |  |  |  |  |  |  |
| Asthma | 4459 (12.2) | 1294 (12.0) | 3165 (12.3) | 0.483 | .487 | .573 |
| Chronic obstructive pulmonary disease | 865 (2.4) | 243 (2.3) | 622 (2.4) | 0.749 | .387 | .465 |
| Lung cancer | 6 (0.0) | 3 (0.0) | 3 (0.0) | 0.429 | .512 | .597 |
| Non melanoma skin cancer | 350 (1.0) | 96 (0.9) | 254 (1.0) | 0.617 | .432 | .514 |
| Stomach cancer | 2 (0.0) | 0 (0.0) | 2 (0.0) | 0.019 | .890 | .931 |
| Oesophagus cancer | 1 (0.0) | 1 (0.0) | 0 (0.0) | 0.203 | .653 | .727 |
| Colon cancer | 5 (0.0) | 0 (0.0) | 5 (0.0) | 0.912 | .339 | .417 |
| Rectal cancer | 11 (0.0) | 4 (0.0) | 7 (0.0) | 0.029 | .865 | .914 |
| Prostate cancer | 145 (0.4) | 64 (0.6) | 81 (0.3) | 14.4 | <.001 | <.001 |
| Ovarian cancer | 20 (0.1) | 0 (0.0) | 20 (0.1) | 7.01 | .008 | .011 |
| Breast cancer | 326 (0.9) | 23 (0.2) | 303 (1.2) | 78.5 | <.001 | <.001 |
| Uterus cancer | 32 (0.1) | 1 (0.0) | 31 (0.1) | 9.47 | .002 | .003 |
| Parkinsons disease | 20 (0.1) | 6 (0.1) | 14 (0.1) | 0.001 | .999 | .999 |
| Fractures | 583 (1.6) | 195 (1.8) | 388 (1.5) | 4.3 | .038 | .051 |
| Cataracts | 777 (2.1) | 207 (1.9) | 570 (2.2) | 2.93 | .087 | .113 |
| Glaucoma | 378 (1.0) | 121 (1.1) | 257 (1.0) | 1.06 | .303 | .381 |
| Physical measurements |  |  |  |  |  |  |
| Body mass index, mean (SE^a^), (kg/m^3^) | 26.6 (0.0) | 27.9 (0.0) | 26 (0.0) | 38.7 | <.001 | <.001 |
| Weight, mean (SE), (kg) | 76.8 (0.1) | 87.1 (0.1) | 72.4 (0.1) | 93.9 | <.001 | <.001 |
| Waist circumference, mean (SE), (cm) | 88 (0.1) | 95.1 (0.1) | 85 (0.1) | 76 | <.001 | <.001 |
| Hip circumference, mean (SE), (cm) | 102.2 (0.0) | 104.6 (0.1) | 101.2 (0.0) | 37.9 | <.001 | <.001 |
| Standing height, mean (SE), (cm) | 169.7 (0.0) | 176.8 (0.1) | 166.7 (0.1) | 115 | <.001 | <.001 |
| SBP, mean (SE), (mmHg) | 135.1 (0.1) | 139.1 (0.2) | 133.5 (0.1) | 27.6 | <.001 | <.001 |
| DBP, mean (SE), (mmHg) | 81.4 (0.1) | 83.5 (0.1) | 80.6 (0.1) | 24.8 | <.001 | <.001 |
| Pulse rate, mean (SE), (bpm) | 67.7 (0.1) | 65.2 (0.1) | 68.8 (0.1) | -29.1 | <.001 | <.001 |
| Arterial stiffness index, mean (SE) | 9.1 (0.0) | 9.7 (0.0) | 8.9 (0.0) | 14.5 | <.001 | <.001 |
| Reflection index, mean (SE) | 67.8 (0.2) | 72.4 (0.3) | 65.8 (0.3) | 14.9 | <.001 | <.001 |
| Trunk fat percentage, mean (SE), (%) | 29.9 (0) | 28 (0.1) | 30.7 (0) | -30.5 | <.001 | <.001 |
| Body fat percentage, mean (SE), (%) | 30 (0) | 26.3 (0.1) | 31.6 (0) | -60.6 | <.001 | <.001 |
| Basal metabolic rate, mean (SE), (KJ) | 6636.4 (7) | 7821.5 (10.8) | 6141 (6.8) | 132 | <.001 | <.001 |
| Forced vital capacity, mean (SE), (litres) | 4 (0.0) | 4.6 (0.0) | 3.7 (0.0) | 77.2 | <.001 | <.001 |
| Forced expiratory volume in 1-second, mean (SE), (litres) | 3 (0.0) | 3.5 (0.0) | 2.8 (0.0) | 71.8 | <.001 | <.001 |
| Forced expiratory volume in 1-second / forced vital capacity ratio, mean (SE), (%) | 0.4 (0.0) | 0.3 (0.0) | 0.4 (0.0) | -4.08 | <.001 | <.001 |
| Family history, n (%) |  |  |  |  |  |  |
| Alzheimer's disease / dementia | 4310 (11.8) | 1180 (11.0) | 3130 (12.2) | 10.2 | .001 | .002 |
| Bowel cancer | 3506 (9.6) | 1023 (9.5) | 2483 (9.7) | 0.15 | .699 | .759 |
| Breast cancer | 2826 (7.7) | 819 (7.6) | 2007 (7.8) | 0.336 | .562 | .648 |
| Chronic bronchitis / emphysema | 4931 (13.5) | 1302 (12.1) | 3629 (14.1) | 25.7 | <.001 | <.001 |
| Diabetes | 6166 (16.9) | 1745 (16.2) | 4421 (17.2) | 4.87 | .027 | .037 |
| Heart disease | 15307 (42.0) | 4243 (39.5) | 11064 (43.0) | 39 | <.001 | <.001 |
| High blood pressure | 15995 (43.9) | 4572 (42.5) | 11423 (44.4) | 10.8 | .001 | .002 |
| Lung cancer | 3832 (10.5) | 1121 (10.4) | 2711 (10.5) | 0.09 | .764 | .822 |
| Parkinson's disease | 1389 (3.8) | 363 (3.4) | 1026 (4.0) | 7.59 | .006 | .008 |
| Severe depression | 3540 (9.7) | 960 (8.9) | 2580 (10.0) | 10.4 | .001 | .002 |
| Stroke | 8953 (24.6) | 2555 (23.8) | 6398 (24.9) | 4.96 | .026 | .035 |
| Medication, n (%) |  |  |  |  |  |  |
| Drugs used in diabetes | 554 (1.5) | 193 (1.8) | 361 (1.4) | 7.52 | .006 | .009 |
| Vitamins | 2468 (6.8) | 574 (5.3) | 1894 (7.4) | 48.9 | <.001 | <.001 |
| Antithrombotic agents | 241 (0.7) | 134 (1.2) | 107 (0.4) | 78.4 | <.001 | <.001 |
| Antihemorrhagics | 88 (0.2) | 10 (0.1) | 78 (0.3) | 13.1 | <.001 | <.001 |
| Antihypertensives | 313 (0.9) | 162 (1.5) | 151 (0.6) | 74.3 | <.001 | <.001 |
| Peripheral vasodilators | 4 (0.0) | 1 (0.0) | 3 (0.0) | 0 | .999 | .999 |
| Vasoprotectives | 1 (0.0) | 0 (0.0) | 1 (0.0) | 0 | .999 | .999 |
| Beta blocking agents | 1452 (4.0) | 625 (5.8) | 827 (3.2) | 133 | <.001 | <.001 |
| Calcium channel blockers | 592 (1.6) | 239 (2.2) | 353 (1.4) | 33.8 | <.001 | <.001 |
| Agents acting on the renin-angiotensin system | 3848 (10.6) | 1524 (14.2) | 2324 (9.0) | 212 | <.001 | <.001 |
| Lipid modifying agents | 5529 (15.2) | 1904 (17.7) | 3625 (14.1) | 76.9 | <.001 | <.001 |
| Anti-inflammatory and antirheumatic products | 7570 (20.8) | 2032 (18.9) | 5538 (21.5) | 31.7 | <.001 | <.001 |
| Statins | 5529 (15.2) | 1904 (17.7) | 3625 (14.1) | 76.9 | <.001 | <.001 |
| Glucocorticoids | 25 (0.1) | 3 (0.0) | 22 (0.1) | 2.88 | .090 | .115 |
| Blood assays |  |  |  |  |  |  |
| Hematocrit percentage, mean (SE), (%) | 13.4 (0.0) | 13.4 (0.0) | 13.4 (0.0) | 55.6 | <.001 | <.001 |
| Red blood cell distribution width, mean (SE), (%) | 250.2 (0.3) | 237.9 (0.5) | 255.4 (0.4) | -1.9 | .073 | .075 |
| Platelet count, mean (SE), (10^9^) | 9.3 (0.0) | 9.3 (0.0) | 9.3 (0.0) | -27.2 | <.001 | <.001 |
| Mean platelet volume, mean (SE), (fL) | 16.5 (0.0) | 16.6 (0.0) | 16.5 (0.0) | 0.501 | .616 | .697 |
| Platelet distribution width, mean (SE), (%) | 13.4 (0.0) | 13.4 (0.0) | 13.4 (0.0) | 15.1 | <.001 | <.001 |
| White blood cell count, mean (SE), (10^9^) | 6.6 (1.8) | 6.5 (2.1) | 6.6 (1.7) | -3.77 | <.001 | <.001 |
| Neutrophil count, mean (SE), (10^9^) | 4 (0.0) | 4 (0.0) | 4 (0.0) | -4.68 | <.001 | <.001 |
| Lymphocyte percentage, mean (SE), (%) | 29.4 (0.0) | 28.9 (0.1) | 29.6 (0.0) | -7.57 | <.001 | <.001 |
| Monocyte percentage, mean (SE), (%) | 7.2 (0.0) | 7.6 (0.0) | 7 (0.0) | 19.3 | <.001 | <.001 |
| Albumin, mean (SE), (g/L) | 45.4 (0.0) | 45.6 (0.0) | 45.3 (0.0) | 9.03 | <.001 | <.001 |
| Alanine aminotransferase, mean (SE), (U/L) | 23.1 (0.1) | 26.3 (0.2) | 21.7 (0.1) | 26.8 | <.001 | <.001 |
| Aspartate aminotransferase, mean (SE), (U/L) | 25.8 (0.1) | 27.6 (0.1) | 25 (0.1) | 20.7 | <.001 | <.001 |
| Gamma glutamyltransferase, mean (SE), (U/L) | 33.7 (0.2) | 39.3 (0.4) | 31.4 (0.2) | 18.8 | <.001 | <.001 |
| Direct bilirubin, mean (SE), (μmol/L) | 1.9 (0.0) | 2 (0.0) | 1.8 (0.0) | 21.4 | <.001 | <.001 |
| Creatinine, mean (SE), (μmol/L) | 72.2 (0.1) | 78.8 (0.1) | 69.4 (0.1) | 60.9 | <.001 | <.001 |
| Urate, mean (SE), (μmol/L) | 303.1 (0.4) | 341.9 (0.7) | 286.9 (0.5) | 63.2 | <.001 | <.001 |
| C-reactive protein, mean (SE), (mg/L) | 2 (0.0) | 2.1 (0.0) | 2 (0.0) | 0.428 | .667 | .734 |
| Glucose, mean (SE), (mmol/L) | 5 (0.0) | 5 (0.0) | 5 (0.0) | 2.68 | .006 | .010 |
| Glycated hemoglobin, mean (SE), (mmol/mol) | 35 (0.0) | 35 (0.1) | 35 (0.0) | 0.569 | .557 | .650 |
| Cholesterol, mean (SE), (mmol/L) | 5.7 (0.0) | 5.6 (0.0) | 5.8 (0.0) | -18.3 | <.001 | <.001 |
| HDL cholesterol, mean (SE), (mmol/L) | 1.5 (0.0) | 1.3 (0.0) | 1.5 (0.0) | -45.7 | <.001 | <.001 |
| Triglycerides, mean (SE), (mmol/L) | 1.6 (0.0) | 1.8 (0.0) | 1.6 (0.0) | 19.2 | <.001 | <.001 |
| LDL direct, mean (SE), (mmol/L) | 3.6 (0.0) | 3.5 (0.0) | 3.6 (0.0) | -7.8 | <.001 | <.001 |
| Lipoprotein A, mean (SE), (nmol/L) | 44.2 (0.3) | 44.9 (0.6) | 43.9 (0.4) | 1.46 | .139 | .182 |
| Cystatin C, mean (SE), (mg/L) | 0.9 (0.0) | 0.9 (0.0) | 0.9 (0.0) | 25 | <.001 | <.001 |
| Sex hormone-binding globulin, mean (SE), (nmol/L) | 52.2 (0.2) | 41.2 (0.2) | 56.8 (0.2) | -53.9 | <.001 | <.001 |
| Testosterone, mean (SE), (nmol/L) | 6.9 (0.0) | 10.9 (0.0) | 5 (0.0) | 93.1 | <.001 | <.001 |

a: *P* values were adjusted by false discovery rate.

B: SE: standard error.

**Table S5. Association between Phenotypes of Cardiac Function and the Risk of Stroke Subtypes.**

| **Phenotype 2 versus 1** | **HR^a^ (95% CI^b^)** | ***P* value** |
| --- | --- | --- |
| Subarachnoid hemorrhage |  |  |
| Model 1^c^ | 0.656 (0.336-1.283) | .218 |
| Model 2^d^ | 0.557 (0.241-1.290) | .172 |
| Model 3^e^ | 0.614 (0.261-1.444) | .264 |
| Intracerebral hemorrhage |  |  |
| Model 1 | 0.626 (0.374-1.049) | .076 |
| Model 2 | 0.505 (0.268-0.951) | .034 |
| Model 3 | 0.557 (0.292-1.063) | .076 |
| Other nontraumatic intracranial hemorrhage |  |  |
| Model 1 | 0.392 (0.198-0.777) | .007 |
| Model 2 | 0.391 (0.170-0.901) | .028 |
| Model 3 | 0.408 (0.175-0.95) | .038 |
| Cerebral infarction |  |  |
| Model 1 | 0.580 (0.462-0.729) | <.001 |
| Model 2 | 0.697 (0.530-0.916) | .010 |
| Model 3 | 0.717 (0.544-0.944) | .018 |
| Stroke, not specified as hemorrhage or infarction |  |  |
| Model 1 | 0.540 (0.287-1.016) | .056 |
| Model 2 | 0.568 (0.250-1.287) | .175 |
| Model 3 | 0.556 (0.244-1.268) | .163 |

a:CI: confidence interval.

b: HR: hazard ratio.

c: Model 1 was an unadjusted model.

d: Model 2 was adjusted for age at recruitment, gender, Townsend deprivation index at recruitment, smoking status, alcohol intake frequency, systolic blood pressure, diastolic blood pressure, body mass index.

e: Model 3 was additionally adjusted for atrial fibrillation, type 2 diabetes, coronary heart disease, heart failure, anti-hypertensive drugs and statins.

**Table S6. Variable Importance of the Selected Covariates.**

| **Covariates** | **Attribute Importance** |
| --- | --- |
| Basal metabolic rate | 0.185 |
| Standing height | 0.142 |
| Gender | 0.138 |
| Testosterone | 0.136 |
| Weight | 0.116 |
| Waist circumference | 0.074 |
| Forced vital capacity | 0.073 |
| Forced expiratory volume in 1-second | 0.064 |
| Creatinine | 0.061 |
| Urate | 0.058 |
| Body fat percentage | 0.054 |
| Hematocrit percentage | 0.046 |
| Sex hormone-binding globulin | 0.041 |
| High-density lipoprotein cholesterol | 0.031 |
| Gamma glutamyltransferase | 0.025 |
| Hip circumference | 0.025 |
| Body mass index | 0.025 |
| Alanine aminotransferase | 0.023 |
| Pulse rate | 0.016 |
| Aspartate aminotransferase | 0.015 |
| Systolic blood pressure | 0.014 |
| Direct bilirubin | 0.013 |
| Trunk fat percentage | 0.013 |
| Cystatin C | 0.012 |
| Platelet count | 0.011 |
| Monocyte percentage | 0.009 |
| Diastolic blood pressure | 0.008 |
| Triglycerides | 0.007 |
| Alcohol intake frequency | 0.006 |
| Cholesterol | 0.005 |
| Platelet distribution width | 0.003 |
| Agents acting on the renin-angiotensin system | 0.003 |
| Coronary heart disease | 0.002 |
| Major adverse cardiac events | 0.002 |
| Atrial fibrillation | 0.002 |
| Forced expiratory volume in 1-second / forced vital capacity ratio | 0.002 |
| Beta blocking agents | 0.002 |
| Ethnic background | 0.001 |
| Breast cancer | 0.001 |
| Smoking status | 0.001 |
| Albumin | 0.001 |
| Antihypertensives | 0.001 |
| Antithrombotic agents | 0.001 |
| Lipid modifying agents | 0.001 |
| Statins | 0.001 |
| Age at recruitment | 0.001 |
| Lymphocyte percentage | 0.001 |
| Vitamins | 0.001 |
| Family history – Heart disease | 0.001 |
| Low-density lipoprotein direct | 0.001 |
| Anti-inflammatory and antirheumatic products | 0.000 |
| Overall health rating | 0.000 |
| Calcium channel blockers | 0.000 |
| Heart failure | 0.000 |
| White blood cell | 0.000 |
| Family history – Chronic bronchitis and emphysema | 0.000 |
| Uterus cancer | 0.000 |
| Antihemorrhagics | 0.000 |
| Type 2 diabetes | 0.000 |
| Prostate cancer | 0.000 |
| Ovarian cancer | 0.000 |
| Family history – High blood pressure | 0.000 |
| Family history – Dementia | 0.000 |
| Family history – Severe depression | 0.000 |
| Family history – Parkinsons disease | 0.000 |
| Drugs used in diabetes | 0.000 |
| Family history – Stroke | 0.000 |
| Family history – Diabetes | 0.000 |
| Fractures | 0.000 |
| Townsend deprivation index | 0.000 |
| Neutrophil count | 0.000 |
| Glucose | 0.000 |

**Table S7. Model Evaluation and Validation with Sensitivity and Specificity.**

|  | **Training set** | | **Validation set** | |
| --- | --- | --- | --- | --- |
| **Models** | Sensitivity^a^ | Specificity | Sensitivity | Specificity |
| K-nearest neighbor | 1.000 (1.000-1.000) | 1.000 (1.000-1.000) | 0.868 (0.858-0.876) | 0.637 (0.615-0.656) |
| Logistic regression | 0.879 (0.875-0.883) | 0.604 (0.593-0.614) | 0.880 (0.871-0.889) | 0.611 (0.589-0.631) |
| Support vector machine | 0.891 (0.887-0.896) | 0.566 (0.556-0.577) | 0.896 (0.887-0.904) | 0.575 (0.553-0.595) |
| Random forest | 0.894 (0.890-0.898) | 0.702 (0.692-0.711) | 0.870 (0.860-0.879) | 0.647 (0.625-0.667) |
| Light gradient boosting machine | 0.873 (0.869-0.877) | 0.659 (0.649-0.669) | 0.871 (0.861-0.880) | 0.647 (0.626-0.666) |
| eXtreme Gradient Boosting | 0.866 (0.861-0.871) | 0.640 (0.630-0.650) | 0.872 (0.863-0.881) | 0.638 (0.617-0.658) |
| artificial neural networks | 0.858 (0.865-0.874) | 0.650 (0.616-0.636) | 0.863 (0.867-0.885) | 0.656 (0.608-0.651) |

a: Presented as value (95% confidence interval).

**Table S8. Model Evaluation and Validation with Precision, F1 score and Balanced accuracy.**

|  | **Training set** | | | **Validation set** | | |
| --- | --- | --- | --- | --- | --- | --- |
| **Models** | Precision^a^ | F1 score | Balanced accuracy | Precision | F1 score | Balanced accuracy |
| K-nearest neighbor | 1.000 (1.000-1.000) | 1.000 (1.000-1.000) | 1.000 (1.000-1.000) | 0.853 (0.842-0.863) | 0.860 (0.852-0.867) | 0.752 (0.740-0.763) |
| Logistic regression | 0.841 (0.836-0.846) | 0.859 (0.856-0.863) | 0.741 (0.736-0.746) | 0.846 (0.835-0.856) | 0.863 (0.855-0.870) | 0.745 (0.733-0.756) |
| Support vector machine | 0.830 (0.825-0.835) | 0.860 (0.856-0.864) | 0.729 (0.723-0.734) | 0.837 (0.826-0.847) | 0.865 (0.857-0.872) | 0.735 (0.723-0.746) |
| Random forest | 0.877 (0.872-0.882) | 0.885 (0.882-0.889) | 0.798 (0.793-0.803) | 0.857 (0.846-0.866) | 0.863 (0.855-0.870) | 0.759 (0.747-0.770) |
| Light gradient boosting machine | 0.859 (0.854-0.864) | 0.866 (0.863-0.870) | 0.766 (0.761-0.771) | 0.857 (0.847-0.866) | 0.864 (0.856-0.871) | 0.759 (0.747-0.769) |
| eXtreme Gradient Boosting | 0.851 (0.846-0.856) | 0.859 (0.855-0.862) | 0.753 (0.747-0.758) | 0.854 (0.844-0.864) | 0.863 (0.856-0.870) | 0.755 (0.744-0.766) |
| artificial neural networks | 0.854 (0.842-0.852) | 0.856 (0.855-0.862) | 0.750 (0.744-0.755) | 0.859 (0.841-0.862) | 0.861 (0.856-0.871) | 0.753 (0.742-0.765) |

a: Presented as value (95% confidence interval).

**Reference**

1. Raisi-Estabragh Z, Harvey NC, Neubauer S, Petersen SE. Cardiovascular magnetic resonance imaging in the UK Biobank: a major international health research resource. Eur Heart J Cardiovasc Imaging 2021 Feb 22;22(3):251–258. PMID:33164079

2. Petersen SE, Matthews PM, Francis JM, Robson MD, Zemrak F, Boubertakh R, Young AA, Hudson S, Weale P, Garratt S, Collins R, Piechnik S, Neubauer S. UK Biobank’s cardiovascular magnetic resonance protocol. J Cardiovasc Magn Reson 2016 Feb 1;18:8. PMID:26830817

3. Bai W, Sinclair M, Tarroni G, Oktay O, Rajchl M, Vaillant G, Lee AM, Aung N, Lukaschuk E, Sanghvi MM, Zemrak F, Fung K, Paiva JM, Carapella V, Kim YJ, Suzuki H, Kainz B, Matthews PM, Petersen SE, Piechnik SK, Neubauer S, Glocker B, Rueckert D. Automated cardiovascular magnetic resonance image analysis with fully convolutional networks. J Cardiovasc Magn Reson 2018 Sept 14;20(1):65. PMID:30217194

4. Hann E, Popescu IA, Zhang Q, Gonzales RA, Barutçu A, Neubauer S, Ferreira VM, Piechnik SK. Deep neural network ensemble for on-the-fly quality control-driven segmentation of cardiac MRI T1 mapping. Med Image Anal 2021 July;71:102029. PMID:33831594

5. Kireeva N, Baskin II, Gaspar HA, Horvath D, Marcou G, Varnek A. Generative Topographic Mapping (GTM): Universal Tool for Data Visualization, Structure-Activity Modeling and Dataset Comparison. Mol Inform 2012 Apr;31(3–4):301–312. PMID:27477099

6. Olier I, Vellido A. Advances in clustering and visualization of time series using GTM through time. Neural Netw 2008 Sept;21(7):904–913. PMID:18653311

7. Vellido A, Lisboa P, Meehan K. The generative topographic mapping as a principal model for data visualization and market segmentation: An electronic commerce case study. Int J Comput Syst Signals 2000;1:119–138.

8. Reimer AP, Madigan EA. Veracity in big data: How good is good enough. Health Informatics J 2019 Dec;25(4):1290–1298. PMID:29388495
